# Nutrient uptake under combined drought and salinity stress in hexaploid wheat species

**DOI:** 10.3389/fpls.2025.1682258

**Published:** 2025-11-10

**Authors:** Mohd. Kamran Khan

**Affiliations:** Department of Soil Science and Plant Nutrition, Faculty of Agriculture, Selcuk University, Konya, Türkiye

**Keywords:** abiotic stress, climate change, genetic resources, genetic variation, nutrient profiling, neglected species, salt stress, water stress

## Abstract

Wheat is an important crop that often suffers from combined drought and salinity stress in agricultural fields, which adversely affects its growth, yield, and nutrient uptake. Understanding the response of genotypes to this combined stress is crucial to developing resilient cultivars. Nutrient uptake patterns in plants under stress not only reveal the physiological effects but also reflect their adaptive strategy and tolerance potential. Neglected and underutilized wheat species with high genetic diversity offer a valuable resource to explore different traits under combined stresses. Notably, no study has thoroughly assessed nutrient uptake in different hexaploid wheat species under combined drought and salinity stress. Thus, this study provides new insights into the individual and combined effects of drought and salinity stresses on nutrient uptake and accumulation of 30 hexaploid wheat genotypes of seven different species grown in a hydroponic system. The combined stress had a synergistic negative effect on nutrient accumulation in wheat genotypes as compared to single stresses. While species-based genetic variability was observed in individual stresses, a greater genotypic diversity was noticed under combined stress. A considerable genotypic variation ranging from 33.6% to 62.6% was observed in traits such as root-shoot phosphorus, manganese, zinc, as well as root copper, iron, and dry weight, while traits like shoot calcium, iron, potassium, and dry weight showed lower genotypic variation (8.3% to 25.8%). Among the studied genotypes, *Tc4* (PI 164160, Kanak, India) was the best performing genotype across all three stress conditions, followed by *Ta3* (CItr 17028, CAR 1101, Chile) and *Tsh2* (PI 42013, India). The patterns of nutrient accumulation proposed that combined stress encompasses a complex interaction of multiple stress pathways. The results yielded valuable insights underscoring the significance of nutrient profiling as a critical component of breeding frameworks for climate-resilient wheat.

## 1 Introduction

In the face of climate change, multiple abiotic stresses such as drought, heat, and salinity occur simultaneously in agricultural regions that severely damage crop production and yield (Pandey et al., 2023; Jing et al., 2024). Shifting precipitation regimes, degradation of soil, rising water salinity, and elevated temperatures together intensify the co-occurrence of these stresses in major crop-growing areas. While plants behave specifically towards individual stresses, their responses can be more complex under combined stresses (Pandey et al., 2023). Two or more stresses can act synergistically, where the response can be more severe than the sum of the individual stresses, or antagonistically, where one stress can reduce the effect of the other (Suzuki et al., 2014; Rivero et al., 2022; Zandalinas and Mittler, 2022; Pandey et al., 2023). Since individual stresses trigger distinct signaling pathways that can be contradictory sometimes, their combination can develop novel responses that cannot be interpreted through individual stress-based studies (Zhang and Sonnewald, 2017; Zandalinas et al., 2020).

Drought and salinity are the two major individual abiotic stress conditions that often coexist together in arid and semiarid zones as **combined drought and salinity stress**. Individually, both drought and salinity reduce water uptake, hamper plant water relations and develop osmotic stress that further leads to a decrease in cell turgor and disrupts key physiological processes such as photosynthesis and stomatal conductance (Ors et al., 2021; Angon et al., 2022; Pandey et al., 2023; Khan et al., 2025). A decrease in photosynthetic pigments, gas exchange, and overall plant biomass, along with an increase in the production of reactive oxygen species and oxidative stress, has also been reported in both the stresses (Dugasa et al., 2019; Ibrahim et al., 2019; Begum et al., 2022). Drought stress restricts nutrient uptake by suppressing root development and reducing the efficiency of proteins involved in nutrient absorption (Hu and Schmidhalter, 2005; Bista et al., 2018; Cheraghi et al., 2023). When occur together with drought, salinity often enhances the stress by accumulating more sodium and chloride ions that compete with essential nutrients such as nitrogen, potassium, and calcium at the transport sites, and thus, impairing the nutrient uptake due to ionic imbalance (Hu and Schmidhalter, 2005; Alam et al., 2020; Ors et al., 2021; Pandey et al., 2023).

Under drought and salinity stress, plants stimulate their defense mechanisms such as closure of stomata, accumulation of osmolytes, and production of antioxidants that remain inadequate under combined stress due to disturbance in coordinated root-shoot signaling (Pierik and Testerink, 2014; Hussain et al., 2021; Angon et al., 2022). The combined drought and salinity stress has been reported to be more detrimental with decreased plant growth and physiological efficiency as compared to drought and salinity stress alone (Dugasa et al., 2019, 2021; Argentel-Martínez et al., 2024; Naqi et al., 2024). Despite the well-recognized damage due to combined drought and salinity stress, most studies focused on drought and salinity stress alone. Due to this gap, there is a limited understanding of how plants survive in actual agricultural fields, where they usually face more than one stress at a time. It is not clear that physiological and molecular responses that provide tolerance under individual drought or salinity stress will be enough to confer tolerance under combined stress conditions. Plants tolerant to individual drought and salinity stress may not be resilient to combined drought and salinity stress, emphasizing the need for screening wide germplasm under combined stress (Pandey et al., 2023).

Macronutrients (such as phosphorus, potassium, calcium, and magnesium) and micronutrients (such as iron, zinc, copper, and manganese) play an important role in plant growth and development (Gupta et al., 2024). While macronutrients are needed in larger amounts and are stable, participating in core physiological processes, micronutrients are needed in trace amounts and show greater plasticity under environmental fluctuations (Khan et al., 2022). The nutrient uptake, translocation, and accumulation can drastically change under abiotic stresses such as drought and salinity. These changes in nutrients not only show the effect of stresses in plants but also reflect their capacity to tolerate stress (Hu and Schmidhalter, 2005). Thus, nutrient accumulation profiles under stress conditions can be used as physiological markers for stress tolerance. However, changes in nutrient dynamics under combined drought and salinity stress have not been well explored, especially in wheat.

Wheat is one of the most important cereal crops largely affected by combined drought and salinity stress mostly in arid and semi-arid regions of the world (Dugasa et al., 2019; Fu et al., 2023; Pandey et al., 2023). Hence, there is an urgent need to screen potential wheat germplasm to identify combined drought and salinity-tolerant genotypes that can be grown in such regions and understand the possible mechanisms behind their tolerance. Neglected wheat species can serve as a potential source of tolerance alleles for combined drought and salinity stress (Ahmadi et al., 2020; Ali et al., 2023; Khan et al., 2025). Hence, it is necessary to utilize their inherent genetic diversity and adaptation capacity in marginal environments for crop improvement in breeding programs. However, there are limited studies investigating their performance against modern wheat cultivars under combined stresses. In fact, no study to date has thoroughly examined the effect of combined drought and salinity stress on nutrient accumulation of different hexaploid wheat species.

Thus, filling this research gap, the present study is the first study investigating the changes in nutrient uptake and accumulation of 30 hexaploid wheat genotypes belonging to seven different species under combined drought and salinity stress. Specifically, the study was conducted to answer the following research questions: 1. Does combined stress have a more pronounced effect on nutrient accumulation in wheat genotypes compared with individual drought and salinity stresses? 2. Are neglected/underutilized wheat species more tolerant to combined drought and salinity stress than modern wheat cultivars? 3. Is there any species-specific accumulation of any particular nutrient under combined drought and salinity stress? 4. Are roots more adversely affected than shoots under combined stress in terms of nutrient accumulation? 5. What is the level of genetic variability in nutrient accumulation in root-shoot tissues of hexaploid wheat genotypes under combined drought and salinity stress? The feedback to these questions can offer critical insights into the role of nutrient accumulation in conferring combined drought and salinity tolerance to hexaploid wheat genotypes, which is crucial for the development of resilient cultivars.

## 2 Materials and methods

### 2.1 Plant material

The experimental material consists of 30 hexaploid accessions of seven different species obtained from the National Small Grains Collection (NSGC), USA, and AARI National Gene Bank and Selcuk University, Turkiye. One of the genotypes, C-306, and three of the genotypes, K 9006, KRL-210, and KRL-213, are well-recognized drought and salinity-tolerant bread wheat cultivars, respectively. The details of the genotypes are provided in **Table 1**.

**Table 1:** List of 30 hexaploid wheat genotypes belonging to seven species used in the experiment.

Genotype code	Taxon	Accession No.	Name	Origin
*Ta1*	*Triticum aestivum*	CItr 12261	Diamond II	Sweden
*Ta2*	*Triticum aestivum*	CItr 12272	SD 2259	United States
*Ta3*	*Triticum aestivum*	CItr 17028	CAR 1101	Chile
*Ta4*	*Triticum aestivum*	CItr 17261	Peking No.10	China
*Ta5*	*Triticum aestivum*	PI 211644	111-33	Turkiye
*Ta6*	*Triticum aestivum*	–	C 306	India
*Ta7*	*Triticum aestivum*	–	K 9006	India
*Ta8*	*Triticum aestivum*	–	KRL 210	India
*Ta9*	*Triticum aestivum*	–	KRL 213	India
*Tc1*	*Triticum compactum*	PI 25970	Bola Blanca	Mexico
*Tc2*	*Triticum compactum*	PI 56213	Mocho de Espiga Quadrada	Portugal
*Tc3*	*Triticum compactum*	PI 129528	Sandomierka	Poland
*Tc4*	*Triticum compactum*	PI 164160	Kanak	India
*Tc5*	*Triticum compactum*	PI 352303	T-1456	Australia
*Tc6*	*Triticum compactum*	PI 668170	Jezka Modra Hladka	Czechoslovakia
*Tc7*	*Triticum compactum*	TR 55030	1040	Turkiye
*Ts1*	*Triticum* sp*elta*	CItr 13967	CI 13967	United States
*Ts2*	*Triticum* sp*elta*	PI 192717	Dankowska Granlatka	Poland
*Ts3*	*Triticum* sp*elta*	PI 306550	2943	Romania
*Ts4*	*Triticum* sp*elta*	PI 347873	69Z6.24	Switzerland
*Ts5*	*Triticum* sp*elta*	PI 591891	Asturien	Spain
*Ts6*	*Triticum* sp*elta*	PI 591899	White Spelt	United Kingdom
*Tsh1*	*Triticum* sp*haerococcum*	CItr 17737	CI 17737	United States
*Tsh2*	*Triticum* sp*haerococcum*	PI 42013	–	India
*Tsh3*	*Triticum* sp*haerococcum*	PI 191301	Sahari M.3	Portugal
*Tsh4*	*Triticum* sp*haerococcum*	PI 272580	I-1-3572	Hungary
*Tsh5*	*Triticum* sp*haerococcum*	PI 330556	Echinatum	United Kingdom
*Tt1*	*Triticum timococcum*	–	254217	England
*Tz1*	*Triticum zhukovskyi*	–	296968	Georgia
*Tsc1*	*Triticum caeruleum*	–	191394	Ethiopia

### 2.2 Experimental design and plant harvest

The experiment was conducted in a hydroponic chamber at Selcuk University, Turkiye, with 22 ± 1 °C temperature, 16000 Lx/day light intensity, 45-55% humidity, and 16 hours light and 8 hours dark photoperiod. Initially, for germination, seeds of each genotype were surface sterilized with sodium hypochlorite followed by 3–4 rinses with distilled water and kept in plastic trays in the dark for 4 days. Four-day-old healthy seedlings were transferred to sterile hydroponic pots containing half-strength Hoagland’s solution. After 4 days of growth in half-strength Hoagland’s solution, plants were subjected to Control (half strength Hoagland solution); Drought stress (15% (w/v) PEG 6000) (Sharma et al., 2022); Salinity stress (150 mM NaCl) (Uzair et al., 2022; Tang et al., 2024); and Combined Drought and Salinity stress [(15% (w/v) PEG 6000 and 150 mM NaCl)] treatments for 9 days and each treatment was replicated three times with five plants per replicate. The 9-day seedling stage treatment was chosen as this stage is commonly used for initial screening of tolerant germplasm. The results obtained may serve as a baseline for identifying promising genotypes and can be further evaluated at reproductive and grain-filling stages in future greenhouse and field studies. For salinity stress treatment, salt was added with 50 mM NaCl increment twice daily to make up to 150 mM NaCl salinity stress to avoid osmotic shocks. The nutrient solutions were changed every 2 days, and after 9 days of stress treatments, roots and shoots were harvested for biomass measurement and nutrient analysis.

### 2.3 Biomass measurement and element quantification

Roots and shoots of all the harvested plants were washed with 0.1 N HCl solution and deionized water, and excess water was collected on the blotting papers (Khan et al., 2022). This was followed by air-drying of root-shoot samples at 70 °C in a hot air oven for 72 hrs, and dry weights were estimated. Dried samples were crushed and 0.20-0.30 g of powdered samples were dissolved in 5 ml of 65% HNO3 and 2 ml of 35% H2O2 and digested in a closed microwave accelerating reaction system (Cem Marsxpress, Matthews, NC, USA) succeeded by the estimation of nutrient amount in the stock solution using ICP-OES (Varian, Vista, Palo Alto, CA, USA). The measurement of the elemental concentration was checked by the certified values of the elements in the reference material provided by the National Institute of Standards and Technology (NIST, Gaithersburg, MD, USA).

### 2.4 Statistical analysis

From the obtained ICP-OES data, initially accumulated nutrient content was calculated using the following formula, and the variation in the accumulated content of different nutrients along with biomass under different treatments has been depicted as Bar diagrams.


$$\begin{array}{c} Tissue\;Accumulated\;Nutrient\;Content({\text{µg plant}}^{-1})=\\ \text{Tissue Nutrient concentration}(\%{\text{ or mg kg}}^{-1})\\ \times \;\text{Tissue dry weight}({\text{g plant}}^{-1}) \end{array}$$

In statistical analysis, the obtained biomass and accumulated nutrient data were subjected to two-way analysis of variance (ANOVA) using the Graphpad Prism 9.0 program, where treatments and genotypes were the two factors and the trait observed was the response. The role of genotypes (G) and treatments (T) in the variability in the expression of traits was considered to be highly significant for the values with P < 0.001. The mean differences among the treatments as compared to the Control was calculated for different traits using Dunnett’s multiple comparisons test, and the comparisons with P < 0.001 were considered to be significantly different. The percentage changes in nutrient accumulation under drought, salinity, and combined drought and salinity stress treatments as compared to the Control treatment were calculated using MS Excel 2010. Minitab (version 16, State College, PA, USA) program-based Pearson correlation analysis was used to determine the association between relative changes in nutrient levels and biomass, considering p < 0.05 to be significant correlation. Heatmaps based on Euclidean distance matrix generated from the average linkage method were drawn to understand the clustering of the genotypes based on the relative changes in their nutrient content and biomass under drought, salinity, and combined drought and salinity stress treatments as compared to the Control.

## 3 Results

### 3.1 Analysis of variance

The analysis of variance (ANOVA) revealed a significantly high genotypic variation (ranging from 33.6% to 62.6%) in traits such as root-shoot phosphorus, root-shoot manganese, root copper, root iron, root-shoot zinc, and root dry weight (**Table 2**). In contrast, shoot calcium, root sodium, shoot iron, root-shoot potassium, and shoot dry weight showed significant but lower genotypic variation (8.3% to 25.8%) (**Table 2**). These observations indicate considerable genetic diversity among the studied genotypes in their nutrient uptake and accumulation capacity under variable growth conditions. In addition to genotypic effects, treatments significantly affected all the studied traits, with the greatest effect on root-shoot sodium content (**Table 2**).

**Table 2:** Results of the two-way analysis of variance (ANOVA) for all the evaluated parameters in shoots and roots of 30 hexaploid wheat genotypes grown under Control, Drought stress, Salinity stress, and Combined Drought and Salinity stress treatments.

Studied Traits	Code	% of total variation		P value		Control vs. Drought		Control vs. Salinity		Control vs. Combined	
		Genotypes	Treatment	Genotypes	Treatment	Mean difference	Adjusted p value	Mean difference	Adjusted p value	Mean difference	Adjusted p value
Shoot DW	SDW	23.0	54.0	****	****	0.0059	***	0.0102	****	0.0194	****
Root DW	RDW	46.3	7.6	****	**	-0.0021	**	-0.0009	ns	-0.0002	ns
Shoot Sodium	SNa	6.1	78.1	ns	****	-0.7767	ns	-291.6000	****	-101.5000	****
Root Sodium	RNa	8.3	76.8	*	****	4.2330	ns	-109.2000	****	-88.6500	****
Shoot Calcium	SCa	19.3	53.6	**	****	64.3200	****	103.1000	****	115.0000	****
Root Calcium	RCa	31.1	6.7	ns	*	6.3670	ns	3.7170	ns	22.5100	*
Shoot Phosphorus	SP	33.6	37.5	****	****	103.3000	****	101.2000	****	149.3000	****
Root Phosphorus	RP	51.6	9.6	****	***	8.9170	*	-2.9970	ns	9.5800	*
Shoot Magnesium	SMg	10.6	68.9	ns	****	38.4800	****	51.2600	****	58.4300	****
Root Magnesium	RMg	20.7	39.4	ns	****	9.7800	****	12.8900	****	15.3200	****
Shoot Potassium	SK	25.8	57.4	****	****	546.0000	****	1172.0000	****	1305.0000	****
Root Potassium	RK	22.6	50.1	***	****	-37.8300	ns	158.4000	****	164.7000	****
Shoot Iron	SFe	26.4	33.7	**	****	0.8664	***	1.2160	****	1.8670	****
Root Iron	RFe	40.9	23.8	****	****	10.6200	****	1.5360	ns	10.2700	****
Shoot Manganese	SMn	38.4	32.5	****	****	0.4036	****	0.6477	****	0.7841	****
Root Manganese	RMn	36.8	28.1	****	****	0.3011	****	0.2982	****	0.4990	****
Shoot Copper	SCu	22.6	25.4	ns	****	0.1919	*	0.3214	****	0.4275	****
Root Copper	RCu	62.6	6.2	****	**	0.8494	**	0.7055	*	0.8403	**
Shoot Zinc	SZn	38.5	35.2	****	****	1.2300	****	1.4680	****	1.8180	****
Root Zinc	RZn	52.9	14.8	****	****	0.9734	****	0.7785	***	1.1230	****

Significant differences are depicted as **** for p < 0.0001; *** for p < 0.0005; ** for p < 0.005; * for p < 0.05; ns, non-significant.

### 3.2 Growth

For shoot growth, all three stresses, drought, salinity, and combined drought and salinity stress, significantly affected the dry weights as compared to the Control treatment (**Table 2**). Drought and salinity stress, individually or in combination, had an overall negative effect on shoot dry weights (**Table 3**, **Supplementary Table S1**, **Supplementary Figure S1**). At an individual level, the effects of drought stress were similar to those of salinity stress (**Table 3**). However, the shoot growth inhibition was more severe in combined drought and salinity stress as compared to drought and salinity stress alone (**Table 3**, **Supplementary Table S1**). Though most of the genotypes showed severe reductions under combined drought and salinity stress as compared to the Control, a significant increase was observed in the *Tc4* genotype (+6%), and two of the genotypes, *Ts3* and *Tsh3*, showed moderate reduction (< 30%) (**Table 3**). Under drought stress alone, *Tc4* showed the highest increase of 62% in SDW, followed by *Ts6* and *Tsh5*. Similar to the other two stress types, in salinity stress as well, *Tc4* was the top-performing genotype with a 13% increase in SDW, succeeded by *Tc3* and *Tsh2*.

**Table 3:** The relative changes (in percentage) in the shoot and root biomass and accumulation of sodium (Na), and calcium (Ca) in 30 hexaploid wheat genotypes in drought (D), salinity (S) and combined drought and salinity (D+S) stress treatments as compared to Control conditions.

Genotype code	Shoot DW			Root DW			Accum shoot Na			Accum root Na			Accum shoot Ca			Accum root Ca		
	D	S	D+S	D	S	D+S	D	S	D+S	D	S	D+S	D	S	D+S	D	S	D+S
*Ta1*	-32	-23	-45	-24	28	-6	-26	5023	2666	-36	943	789	-42	-64	-56	-31	-18	-39
*Ta2*	-28	-11	-46	-4	46	17	-39	152	880	-24	902	979	-35	-95	-51	-17	-16	-30
*Ta3*	-4	4	-23	31	29	27	-4	4059	1889	-23	973	866	-3	-36	-31	40	3	-7
*Ta4*	-32	-35	-49	-15	-14	-25	-35	4816	1415	-41	505	514	-47	-66	-64	-25	-36	-36
*Ta5*	-14	-25	-63	57	12	-36	-22	5187	948	-73	301	108	-60	-83	-90	-54	-93	-93
*Ta6*	-42	-47	-43	-19	-9	20	-60	6695	2804	-22	1123	2659	-63	-45	-47	-30	97	108
*Ta7*	-24	-28	-51	-15	16	10	-62	5094	2228	-74	1018	1341	-59	-49	-53	-5	185	151
*Ta8*	-33	-43	-48	7	14	75	-31	2690	2891	27	3145	4995	-57	-41	-43	-20	156	142
*Ta9*	-27	-36	-61	2	11	5	-33	5612	2320	-51	1160	1368	-54	-40	-49	-45	67	63
*Tc1*	-30	-21	-49	25	32	-2	-29	6396	1458	-66	670	378	-62	-80	-81	-44	-90	-89
*Tc2*	-13	-30	-39	63	2	46	13	4375	1395	-76	187	227	-56	-89	-66	-46	-93	-85
*Tc3*	24	9	-40	100	16	30	37	8601	887	-39	651	425	-17	-63	-77	-29	-93	-88
*Tc4*	62	13	6	133	28	58	20	5685	1635	-98	557	487	-1	-67	-69	-81	-62	-74
*Tc5*	-45	-20	-48	-36	6	-28	-57	3856	808	-85	345	153	-54	-70	-76	-27	-69	-78
*Tc6*	-15	-29	-62	73	30	6	30	4428	1392	-3	699	402	-35	-80	-84	-11	-82	-91
*Tc7*	-9	-26	-38	49	-12	14	241	6853	2655	253	3668	4098	16	-42	-70	209	118	-52
*Ts1*	6	-37	-52	95	7	17	106	5770	1058	8	1492	1038	29	-68	-70	97	-84	-84
*Ts2*	-46	-28	-51	-19	-6	-28	-6	2756	801	-11	740	335	-36	-80	-80	48	-78	-82
*Ts3*	-11	-43	-68	13	-28	-36	113	3360	1306	-39	603	169	0	-81	-86	37	-85	-80
*Ts4*	-11	-23	-62	70	38	-4	19	4019	1092	1	1274	555	1	-78	-75	126	-77	-71
*Ts5*	-21	-35	-61	8	13	-6	92	5026	904	-76	617	337	-58	-71	-76	-77	-86	-76
*Ts6*	31	-8	-45	115	-2	-33	17	12102	5034	614	5298	2516	37	-6	3	43	225	249
*Tsh1*	4	-10	-59	29	38	23	166	11572	3974	190	3244	3105	-28	-21	-74	22	379	51
*Tsh2*	11	5	-39	93	44	12	174	8672	4816	187	4919	2489	-15	2	-56	91	502	43
*Tsh3*	-18	-6	-27	68	40	35	134	13733	6807	88	4400	3519	-35	-14	-66	-13	512	-38
*Tsh4*	-1	0	-48	50	51	-10	149	13657	5891	73	2162	1553	-15	-27	-72	-19	174	-43
*Tsh5*	27	-19	-37	162	26	35	108	10047	4211	385	3853	3840	-5	-24	-64	84	240	-46
*Tt1*	-32	-52	-54	-44	-48	-10	-27	5320	3062	-52	1011	-39	-64	-49	-54	-68	14	-89
*Tz1*	-10	-35	-58	16	44	-24	-54	4374	2939	-16	1381	1278	-53	-43	-52	-60	30	1
*Tsc1*	-15	-44	-51	10	-5	4	64	4983	1505	-58	1147	791	-53	-68	-75	-21	21	-22

In case of root growth, only drought stress had a significant effect on dry weights, while salinity and combined stress did not affect them significantly (**Table 2**). In drought stress, *Tsh5* and *Tc4* showed the highest increase of 162% and 133% respectively, in root dry weights (RDW) as compared to Control (**Table 3**, **Supplementary Figure S2**). Additionally, concerning dry weights, shoots were more affected than roots, either by individual drought and salinity stress or combined drought and salinity stress (**Table 3**).

### 3.3 Sodium uptake and accumulation

Compared with the Control treatment, drought stress did not significantly affect the root and shoot sodium content, while salinity stress and combined stress significantly affected them (**Table 2**). As expected, the sodium content of all the genotypes increased under salinity stress and combined stress as compared to the Control due to the external supply of 150 mM NaCl (**Table 3**, **Supplementary Table S1**, **Supplementary Figure S3**). There was a great variation in sodium accumulation in shoots and roots under both salinity and combined stress.

In shoots, salinity stress led to a maximum and minimum increase of more than 13000% and 152% in the sodium content of *Tsh3* and *Ta2*, respectively. However, combined stress showed a maximum and minimum increase of more than 6000% and 800% in *Tsh3* and *Ta2*, respectively (**Table 3**). Interestingly, other than *Ta2* and *Ta8*, for all the genotypes combined stress had a diminishing effect on sodium accumulation as compared to salinity stress alone. Similarly, in roots, while salinity stress led to a maximum and minimum increase of more than 5000% in *Ts6* and 187% in *Tc2*, respectively, combined stress showed a maximum increase of more than 4000% in *Tc7* and a minimum decrease 39% in *Tt1*, respectively, in the sodium content (**Table 3**, **Supplementary Figure S4**). Similar to shoots, in roots also, combined stress had a diminishing effect on sodium uptake as compared to salinity stress for most of the genotypes (**Table 3**, **Supplementary Table S1**).

### 3.4 Calcium uptake and accumulation

In case of shoots, all three stresses, drought, salinity, and combined drought and salinity stress significantly affected the calcium content as compared to the Control treatment (**Table 2**). Other than a few genotypes, drought and salinity stress individually or in combination had an overall negative effect on calcium content (**Table 3**, **Supplementary Table S1**). Although it was a mixed genotype-dependent response, salinity and combined stress seem to have a more negative effect on calcium accumulation than drought stress (**Supplementary Figure S5**). Though most of the genotypes showed severe reductions under combined stress as compared to the Control, genotype *Ts6* showed an increase of 3% and genotype *Ta3* showed a moderate reduction of 30% (**Table 3**, **Supplementary Table S1**). Under drought stress alone, *Ts6* showed the highest increase of 37% in calcium content, followed by *Ts1* and *Tc7*. In salinity stress, only one of the studied genotypes, *Tsh2*, showed an increase of 2% in calcium content as compared to Control; all the other genotypes showed a reduction (**Table 3**, **Supplementary Table S1**).

In the case of roots, only combined stress had a significant effect on calcium uptake, while drought and salinity stress did not affect them significantly. In combined stress, *Ts6* and *Ta7* showed the highest increase of 249% and 151% respectively, in calcium uptake as compared to Control (**Table 3**, **Supplementary Table S1**, **Supplementary Figure S6**). Additionally, concerning calcium content, shoots were overall more affected than roots by combined stress.

### 3.5 Phosphorus uptake and accumulation

In case of shoot, all three stresses, drought, salinity, and combined stress significantly affected the phosphorus content as compared to the Control treatment (**Table 2**). Drought and salinity stress, individually or in combination, had an overall negative effect on phosphorus accumulation in shoots (**Table 4**, **Supplementary Table S2**). Although it was a mixed genotype-dependent response, combined stress seems to have a more negative effect on phosphorus accumulation than drought and salinity stresses (**Supplementary Figure S7**). Though most of the genotypes showed severe reductions under combined stress as compared to the Control, a significant increase was observed in the *Tc4* genotype (+48%), and genotype *Tc5* showed a moderate reduction (< 30%) in phosphorus (**Table 4**). Under drought stress alone, *Tc4* and *Ts1* showed the greatest increase of 72% and 8% in phosphorus content. Similar to the other two stress types, in salinity stress as well, *Tc4* was the top performing genotype with 48% increase in phosphorus accumulation, followed by *Ta3* and *Tc5* (**Table 4**).

**Table 4:** The relative changes (in percentage) in the shoot and root accumulation of phosphorus (P), magnesium (Mg), and potassium (K) in 30 hexaploid wheat genotypes in drought (D), salinity (S) and combined drought and salinity (D+S) stress treatments as compared to Control conditions.

Genotype code	Accum shoot P			Accum root P			Accum shoot Mg			Accum root Mg			Accum shoot K			Accum root K		
	D	S	D+S	D	S	D+S	D	S	D+S	D	S	D+S	D	S	D+S	D	S	D+S
*Ta1*	-38	-24	-54	-40	29	-8	-45	-53	-58	-49	-42	-59	-40	-55	-66	-16	-70	-70
*Ta2*	-32	-94	-49	-17	40	27	-31	-95	-48	-30	-30	-38	-27	-97	-48	-9	-63	-59
*Ta3*	-13	19	-32	1	35	19	-17	-18	-36	-4	-33	-40	-15	-18	-35	29	-61	-51
*Ta4*	-33	-47	-57	-32	-33	-38	-53	-74	-64	-35	-62	-58	-41	-68	-66	-15	-78	-79
*Ta5*	-16	-33	-48	2	21	-39	-59	-70	-76	-68	-81	-90	-14	-58	-66	136	-17	-48
*Ta6*	-61	-58	-58	-45	-37	-20	-57	-62	-60	-49	-45	-12	-56	-66	-58	-27	-82	-72
*Ta7*	-64	-39	-67	-49	-5	-26	-60	-45	-63	-47	-16	-17	-46	-49	-69	-33	-71	-66
*Ta8*	-54	-46	-58	-30	-11	30	-58	-54	-66	-42	-15	5	-41	-52	-66	-10	-63	-62
*Ta9*	-49	-32	-60	-41	-24	-30	-53	-55	-68	-34	-18	-7	-45	-55	-71	-26	-80	-81
*Tc1*	-46	-16	-41	-23	47	34	-68	-60	-67	-69	-71	-81	-38	-54	-60	48	-40	-50
*Tc2*	-37	-28	-39	5	-16	55	-54	-68	-66	-68	-89	-75	-20	-67	-47	199	-44	17
*Tc3*	-7	-15	-33	15	7	5	-37	-47	-55	-50	-79	-78	3	-43	-60	157	-34	-63
*Tc4*	72	54	48	-78	122	108	14	-34	-15	-85	-68	-59	148	1	20	-72	-12	-9
*Tc5*	-36	1	-23	-40	29	-26	-64	-63	-58	-77	-77	-82	-37	-44	-49	13	-26	-68
*Tc6*	-30	-14	-47	-6	30	-12	-42	-50	-66	-42	-72	-74	-37	-54	-66	26	-60	-72
*Tc7*	-50	-58	-57	-28	-21	-33	-34	-59	-66	-10	-48	-61	-20	-55	-56	72	-85	-65
*Ts1*	8	-34	-44	47	44	-1	1	-47	-45	54	-45	-41	-9	-69	-69	35	-66	-79
*Ts2*	-35	-10	-31	-21	22	-34	-44	-40	-53	-22	-63	-71	-54	-63	-68	-12	-68	-83
*Ts3*	-5	-23	-61	-11	-15	-56	-17	-64	-76	-43	-77	-88	-11	-59	-76	5	-82	-88
*Ts4*	-33	-44	-61	35	23	-41	-23	-43	-56	54	-33	-69	-36	-63	-72	38	-62	-83
*Ts5*	-11	-14	-44	14	35	-8	-33	-47	-60	-58	-65	-71	-17	-51	-65	38	-62	-67
*Ts6*	-26	-29	-50	1	-4	-65	3	-48	-59	57	-11	-36	7	-51	-68	92	-85	-89
*Tsh1*	-11	-26	-64	15	39	12	-12	-40	-69	15	-6	-50	3	-44	-68	41	-54	-57
*Tsh2*	-9	-2	-39	45	120	4	-24	-22	-55	27	40	-56	10	-17	-49	115	-67	-62
*Tsh3*	-45	-31	-52	2	44	13	-38	-44	-60	-3	51	-39	-31	-48	-60	51	-55	-53
*Tsh4*	-28	-25	-48	-1	12	-18	-18	-38	-61	-22	-21	-75	-2	-37	-55	29	-71	-78
*Tsh5*	-19	-42	-50	62	24	-10	-10	-46	-64	86	3	-53	1	-56	-60	246	-68	-62
*Tt1*	-54	-64	-65	-50	-53	-94	-56	-65	-66	-66	-77	-97	-54	-78	-70	-56	-89	-98
*Tz1*	-53	-42	-70	-32	-3	-59	-48	-48	-69	-36	-37	-49	-27	-55	-67	-13	-72	-89
*Tsc1*	-37	-26	-41	-4	28	12	-55	-47	-62	-73	-75	-79	-37	-57	-58	21	-72	-71

In the case of roots, drought and combined stress had a significant effect on phosphorus uptake, while salinity stress did not affect them significantly. The effect was genotype dependent and not overall positive or negative under both drought and combined stress conditions (**Table 4**, **Supplementary Table S2**, **Supplementary Figure S8**). While in drought stress, *Tsh5* and *Ts1* showed the highest increase of 62% and 47% respectively, as compared to Control, in combined stress, *Tc4* and *Tc2* were the best performing genotypes with 108% and 55% increases in phosphorus accumulation, respectively (**Table 4**). Additionally, concerning phosphorus content, shoots were more affected than roots by combined stress (**Table 4**).

### 3.6 Magnesium uptake and accumulation

Compared with the Control treatment, all three stresses, drought, salinity, and combined stress, significantly affected the root and shoot magnesium content (**Table 2**). Drought and salinity stress, individually or in combination, had an overall negative effect on magnesium accumulation in both roots and shoots (**Table 4**, **Supplementary Table S2**). Combined stress and salinity stress seem to have a more negative effect on magnesium accumulation than drought stress (**Supplementary Figure S9**). In shoots, combined stress had a diminishing effect on magnesium accumulation of all the genotypes, with minimum decrease of 15% in *Tc4*, followed by *Ta3*. While drought stress led to the greatest increase of 14% in *Tc4* and 1% in *Ts1*, respectively, salinity stress showed a minimum decrease of 18% in *Ta3* followed by *Tsh2* (**Table 4**, **Supplementary Figure S10**). Similarly, in roots, combined stress had diminishing effects on the magnesium content of all the genotypes except *Ta8*, which showed an increase of 5% (**Table 4**). While drought stress led to the greatest increase of 86% and 57% in *Tsh5* and *Ts6*, respectively, salinity stress showed an increase of 51% and 40% in genotypes *Tsh3* and *Tsh2*, respectively (**Table 4**). Additionally, concerning magnesium content, shoots were more affected than roots by combined drought and salinity stress (**Table 4**).

### 3.7 Potassium uptake and accumulation

In case of shoot, all three stresses, drought, salinity, and combined stress significantly affected the potassium content as compared to the Control treatment (**Table 2**). Drought and salinity stress, individually or in combination, had an overall negative effect on potassium accumulation in shoots (**Table 4**, **Supplementary Table S2**). Although it was a mixed genotype-dependent response, combined stress seems to have a more negative effect on potassium accumulation than drought and salinity stresses (**Supplementary Figure S11**). Under salinity and combined stress as compared to Control, all the genotypes showed severe reductions, except one genotype, *Tc4*, that showed an increase of 1% and 20%, respectively (**Table 4**). Under drought stress alone, *Tc4* and *Ts1* showed the greatest increase of 72% and 8% in phosphorus content. Similar to the other two stress types, in drought stress as well, *Tc4* showed the greatest increase of 148% followed by *Tsh2* and *Ts6* (**Table 4**).

In the case of roots, salinity and combined stress had a significant effect on potassium accumulation, while salinity stress did not affect them significantly. While *Tc2* and *Tc4* showed the least suppressive effect of combined stress, under salinity stress, the potassium content of *Tc4* and *Ta5* was the least affected (**Table 4**, **Supplementary Table S2**, **Supplementary Figure S12**). Contrary to other macronutrients, the potassium content of roots was more decreased than in shoots under combined stress as compared to the Control treatment.

### 3.8 Uptake and accumulation of micronutrients

The iron accumulation in shoots is significantly affected by all three stresses, drought, salinity, and combined stress, while in roots, it is significantly affected by drought and combined stress only (**Table 2**). Although it was a mixed geno-type-dependent response, combined stress seems to have a more negative effect on iron accumulation than drought and salinity stresses (**Supplementary Figure S13**). In both roots and shoots, combined stress has overall reduced the iron accumulation (**Supplementary Figure S13**, **Supplementary Figure S14**). In shoots, *Tsh5* and *Ts6* were the genotypes with a minimum decrease in iron accumulation under combined stress as compared to Control (**Table 5**, **Supplementary Table S3**). However, in roots, *Tsc1* showed an increase of 27% followed by *Ta2*, which showed a decrease of only 1% under combined drought and salinity stress (**Table 5**). Under drought stress alone, *Ts6* and *Ts4* were the genotypes with maximum iron accumulation in shoots and roots, respectively (**Table 5**).

**Table 5:** The relative changes (in percentage) in the shoot and root accumulation of iron (Fe), manganese (Mn), copper (Cu) and zinc (Zn) in 30 hexaploid wheat genotypes in drought (D), salinity (S) and combined drought and salinity (D+S) stress treatments as compared to Control conditions.

Genotype code	Accum shoot Fe			Accum root Fe			Accum shoot Mn			Accum root Mn			Accum shoot Cu			Accum root Cu			Accum shoot Zn			Accum root Zn		
	D	S	D+S	D	S	D+S	D	S	D+S	D	S	D+S	D	S	D+S	D	S	D+S	D	S	D+S	D	S	D+S
*Ta1*	-28	-22	-30	-67	-12	-66	-50	-52	-59	-35	-29	-42	18	-16	-26	-66	-55	-67	-78	-80	-83	-86	-80	-91
*Ta2*	-7	-80	-37	-42	97	-1	-28	-95	-54	-20	-45	-54	-12	-92	-33	-64	-57	-64	-52	-98	-59	-76	-80	-69
*Ta3*	0	1	-14	-14	26	-19	-9	3	-23	12	-8	-32	9	-13	-10	-23	-46	-50	-19	-10	-39	-29	-56	-49
*Ta4*	-36	-44	-43	-36	-28	-39	-39	-63	-67	12	-43	-53	-34	-57	-50	-66	-78	-77	-79	-86	-85	-87	-90	-87
*Ta5*	-43	-61	-81	-24	13	-48	-35	-59	-60	-31	-64	-64	-64	-81	-86	-60	-88	-88	-52	-60	-71	-69	-87	-100
*Ta6*	-56	-12	-28	-60	-46	-31	-45	-56	-43	-48	-45	-47	-57	-18	-6	-78	-61	47	-68	-54	-67	-46	-46	-11
*Ta7*	-46	-12	-25	-81	-35	-78	-47	-40	-54	-54	-11	-75	-50	7	22	-79	-44	19	-53	-41	-68	-76	-21	-61
*Ta8*	-56	-44	-42	-51	-13	-7	-53	-56	-66	-45	-34	-70	-62	-11	-10	-69	-51	38	-66	-68	-64	-69	-56	-7
*Ta9*	-44	-22	-42	-80	-60	-56	-35	-40	-72	-51	-18	-76	-56	-4	-20	-68	-35	54	-66	-55	-74	-63	-41	-18
*Tc1*	-62	-65	-69	-39	34	-48	-53	-34	-39	-40	-29	-42	-48	-65	-82	-41	-67	-84	-75	-70	-74	-63	-82	-82
*Tc2*	-20	-38	-55	-50	-39	-37	-37	-57	-43	-52	-74	-24	-15	-75	-80	-52	-84	-73	-48	-69	-70	-40	-93	-75
*Tc3*	-1	-32	-56	-7	-3	-47	-22	-48	-34	-10	-34	-49	-40	-62	-70	-40	-81	-77	-14	-62	-67	-82	-100	-100
*Tc4*	27	-5	-37	-83	109	-13	66	-24	38	-78	-37	11	-9	-77	-60	-84	-55	-52	42	24	-16	-69	-55	-68
*Tc5*	-31	-40	-46	-75	-30	-66	-53	-41	-25	-66	-48	-59	-67	-69	-72	-45	-67	-73	-73	-66	-63	-96	-80	-89
*Tc6*	8	-22	-57	-2	146	-26	-47	-57	-58	-48	-62	-61	-15	-56	-83	-8	-59	-72	-35	-32	-63	-100	-68	-68
*Tc7*	27	-31	-61	-62	-40	-78	-9	-40	-38	-53	-75	-70	-2	-18	-61	-36	-40	-78	-17	-55	-54	-19	1	-28
*Ts1*	13	-3	-54	52	157	-44	18	-46	-35	23	-62	-65	96	-64	-62	100	-49	-69	31	-29	-32	82	-32	-69
*Ts2*	-35	-48	-41	17	45	-63	-39	-28	-35	-64	-78	-79	-12	-54	-71	64	-39	-64	-39	-15	-50	-59	22	-76
*Ts3*	26	-41	-69	-43	52	-82	-10	-46	-70	-38	-82	-86	79	-34	-66	-14	-75	-87	-42	-66	-81	-73	-85	-82
*Ts4*	-10	-48	-63	97	94	-58	-6	-28	-45	-13	-64	-75	53	-55	-63	68	-54	-68	-20	-32	-55	63	34	-44
*Ts5*	-33	-38	-60	-22	87	-27	-6	-21	-24	-37	-57	-44	-38	-43	-70	-74	-73	-76	-14	-15	-47	-74	-73	-53
*Ts6*	32	22	-5	-70	-49	-85	35	-25	-41	-61	-77	-86	37	72	50	-7	-34	-78	7	-30	-49	-7	-18	-48
*Tsh1*	-16	-1	-58	-46	3	-18	3	-17	-59	-49	-62	-85	-10	57	-56	14	-21	-65	-26	-27	-69	-47	23	-37
*Tsh2*	-6	21	-35	-55	60	-71	15	30	-33	-15	-35	-69	0	21	-48	-29	85	-68	4	7	-41	53	254	26
*Tsh3*	-34	-6	-46	-39	30	-39	2	12	-21	-41	-34	-61	-40	25	-44	-23	43	-45	-22	-15	-36	-29	51	-31
*Tsh4*	16	16	-28	-23	33	-37	28	19	-28	-31	0	-70	-14	18	-61	-8	-6	-79	24	-12	-53	13	57	-32
*Tsh5*	24	4	-4	-36	-2	-50	54	-8	-30	24	-34	-60	-12	9	1	46	15	-59	7	-26	-38	120	81	-17
*Tt1*	-44	-25	-24	-82	-63	-96	-49	-58	-56	-62	-70	-100	-54	-34	-23	-72	-62	-91	-66	-56	-76	-33	-44	-98
*Tz1*	-15	-22	-38	-77	-38	-81	-32	-33	-67	-53	54	-80	-34	0	-21	-63	-2	-8	-59	-58	-75	-47	-37	-63
*Tsc1*	-42	-36	-48	-53	161	27	-29	-37	-44	-7	32	-57	-47	-50	-53	-62	2	9	-50	-61	-66	-16	-1	-54

Compared with the Control treatment, all three stresses, drought, salinity, and combined drought and salinity, significantly affected the manganese accumulation in both roots and shoots (**Table 2**). Under drought and salinity stress, some of the genotypes showed increased manganese accumulation in both roots and shoots (**Supplementary Figure S15**, **Supplementary Figure S16**). However, under combined drought and salinity stress, reduced accumulation was observed in all the genotypes but one, *Tc4* which showed 38% and 11% increase in shoots and roots, respectively (**Table 5**, **Supplementary Table S3**).

The copper accumulation in both roots and shoots is significantly affected by all three stresses, drought, salinity, and combined stress (**Table 2**). Although it was a mixed genotype-dependent response, combined stress seems to have a more negative effect than drought and salinity stresses on copper accumulation (**Supplementary Figures S17**, **S18**). In shoots, *Ts6* and *Ta7* were the genotypes with a maximum increase of 50% and 22% in copper accumulation under combined stress as compared to the Control (**Table 5**, **Supplementary Table S4**). However, in roots, *Ta9* and *Ta6* showed an increase of 54% and 47% respectively, under combined drought and salinity stress. Under drought stress alone, genotype *Ts1* showed the maximum copper accumulation (96% in shoots and 100% in roots), while in salinity stress, genotypes *Ts6* and *Tsh2* were the ones with maximum shoot and root content (**Table 5**, **Supplementary Table S4**).

Compared with the Control treatment, all three stresses, drought, salinity, and combined significantly affected the zinc accumulation in both roots and shoots. Under combined stress as compared to Control, roots and shoots of all the genotypes showed severe reductions except one genotype, *Tsh2*, which showed an increase of 26% in roots (**Table 5**, **Supplementary Table S4**). Interestingly, in shoots, genotype *Tc4* had the least effect of drought, salinity, and combined stresses on zinc accumulation. In addition, some of the genotypes, such as *Tsh2* and *Tsh5*, were among the ones with the greatest zinc accumulation in both roots and shoots (**Table 5**, **Supplementary Figures S19**, **S20**).

### 3.9 Association between accumulated nutrients and dry weights of studied genotypes under combined drought and salinity stress

The association between relative nutrient accumulation under combined drought and salinity stress, as compared to the Control treatment, was estimated using correlation analysis (**Table 6**). Interestingly, except for magnesium and iron, relative changes of all the other nutrients in shoots were significantly correlated to their relative changes in roots under combined drought and salinity stress (**Table 6**).

**Table 6:** Association between relative accumulated nutrients and dry weights of 30 hexaploid wheat genotypes under combined drought and salinity stress (p < 0.05 was considered to be significant correlation; purple marked columns showed the significant correlation between the corresponding traits).

	SDW	RDW	SNa	RNa	SCa	RCa	SP	RP	SMg	RMg	SK	RK	SFe	RFe	SMn	RMn	SCu	RCu	SZn
RDW	0.59**																		
p-value	**0.001**																		
SNa	**0.23**	**0.15**																	
p-value	**0.233**	**0.418**																	
RNa	**0.20**	0.49**	0.654**																
p-value	**0.285**	**0.006**	**0.000**																
SCa	**0.25**	**0.12**	0.40*	0.37*															
p-value	**0.191**	**0.534**	**0.027**	**0.043**															
RCa	**0.01**	**0.12**	0.44*	0.55**	0.78**														
p-value	**0.967**	**0.525**	**0.015**	**0.001**	**0.000**														
SP	0.72**	**0.30**	**-0.22**	**-0.27**	**-0.15**	**-0.29**													
p-value	**0.000**	**0.107**	**0.234**	**0.155**	**0.426**	**0.122**													
RP	0.65**	0.74**	**-0.09**	**0.08**	**-0.13**	**-0.12**	0.66**												
p-value	**0.000**	**0.000**	**0.625**	**0.661**	**0.501**	**0.516**	**0.000**												
SMg	0.76**	0.40*	**-0.12**	**-0.13**	**0.19**	**-0.08**	0.78**	0.57**											
p-value	**0.000**	**0.029**	**0.525**	**0.479**	**0.305**	**0.677**	**0.000**	**0.001**											
RMg	**0.17**	0.46*	**0.29**	0.62**	0.62**	0.75**	**-0.20**	**0.16**	**0.15**										
p-value	**0.372**	**0.010**	**0.126**	**0.000**	**0.000**	**0.000**	**0.303**	**0.394**	**0.444**										
SK	0.86*	0.47*	**-0.05**	**-0.07**	**0.04**	**-0.14**	0.89**	0.72**	0.79**	**-0.01**									
p-value	**0.000**	**0.009**	**0.809**	**0.709**	**0.838**	**0.447**	**0.000**	**0.000**	**0.000**	**0.957**									
RK	0.58**	0.59**	**-0.11**	**-0.03**	**-0.16**	**-0.19**	0.56**	0.82**	**0.32**	**-0.04**	0.65**								
p-value	**0.001**	**0.001**	**0.554**	**0.886**	**0.414**	**0.304**	**0.001**	**0.000**	**0.081**	**0.830**	**0.000**								
SFe	0.41*	**0.14**	0.49**	**0.30**	0.76**	0.53**	**0.03**	**-0.09**	**0.33**	0.42*	**0.19**	**-0.17**							
p-value	**0.025**	**0.452**	**0.006**	**0.103**	**0.000**	**0.003**	**0.891**	**0.627**	**0.077**	**0.020**	**0.327**	**0.371**							
RFe	**0.24**	0.52**	**-0.16**	**0.08**	**-0.13**	**-0.06**	**0.32**	0.69**	**0.31**	**0.17**	0.37*	0.41*	**-0.12**						
p-value	**0.206**	**0.004**	**0.387**	**0.691**	**0.479**	**0.762**	**0.090**	**0.000**	**0.093**	**0.372**	**0.046**	**0.025**	**0.531**						
SMn	0.80**	**0.36**	**0.09**	**-0.03**	**-0.07**	**-0.23**	0.85**	0.57**	0.79**	**-0.13**	0.81**	0.47**	**0.18**	**0.22**					
p-value	**0.000**	**0.052**	**0.656**	**0.873**	**0.727**	**0.230**	**0.000**	**0.001**	**0.000**	**0.489**	**0.000**	**0.009**	**0.342**	**0.242**					
RMn	0.70**	0.47**	**-0.29**	**-0.22**	**-0.11**	**-0.28**	0.75**	0.81**	0.63**	**-0.02**	0.79**	0.76**	**-0.02**	0.51**	0.64**				
p-value	**0.000**	**0.008**	**0.125**	**0.240**	**0.564**	**0.131**	**0.000**	**0.000**	**0.000**	**0.899**	**0.000**	**0.000**	**0.909**	**0.004**	**0.000**				
SCu	**0.14**	**0.10**	0.44**	0.46**	0.88**	0.82**	**-0.30**	**-0.25**	**0.05**	0.67**	**-0.11**	**-0.30**	0.82**	**-0.20**	**-0.16**	**-0.25**			
p-value	**0.455**	**0.605**	**0.015**	**0.011**	**0.000**	**0.000**	**0.112**	**0.180**	**0.795**	**0.000**	**0.580**	**0.110**	**0.000**	**0.283**	**0.414**	**0.188**			
RCu	**0.00**	**0.35**	**0.09**	0.37*	**0.34**	0.58**	**-0.18**	**0.09**	**-0.04**	0.76**	**-0.05**	**-0.08**	**0.25**	**0.26**	**-0.21**	**-0.03**	0.48**		
p-value	**0.991**	**0.062**	**0.657**	**0.044**	**0.068**	**0.001**	**0.336**	**0.627**	**0.847**	**0.000**	**0.814**	**0.683**	**0.185**	**0.169**	**0.263**	**0.894**	**0.007**		
SZn	0.65**	0.42*	**0.25**	**0.26**	**0.11**	**-0.03**	0.60**	0.44*	0.73**	**0.16**	0.58**	**0.27**	**0.28**	**0.21**	0.82**	**0.35**	**0.04**	**-0.11**	
p-value	**0.000**	**0.022**	**0.192**	**0.167**	**0.555**	**0.891**	**0.000**	**0.014**	**0.000**	**0.409**	**0.001**	**0.147**	**0.139**	**0.255**	**0.000**	**0.061**	**0.818**	**0.578**	
RZn	**0.15**	0.42*	0.59**	0.77**	**0.34**	0.53**	**-0.16**	**0.13**	**0.01**	0.60**	**0.04**	**-0.03**	**0.32**	**0.14**	**0.08**	**-0.14**	**0.36**	0.47**	0.38*
p-value	**0.433**	**0.022**	**0.001**	**0.000**	**0.063**	**0.003**	**0.406**	**0.504**	**0.970**	**0.000**	**0.847**	**0.859**	**0.089**	**0.463**	**0.695**	**0.461**	**0.050**	**0.008**	**0.037**

SDW, Shoot Dry Weight; RDW, Root Dry Weight; SNa, Shoot Sodium; RNa, Root Sodium; SCa, Shoot Calcium; RCa, Root Calcium; SP, Shoot Phosphorus; RP, Root Phosphorus; SMg, Shoot Magnesium; RMg, Root Magnesium; SK, Shoot Potassium; RK, Root Potassium; SFe, Shoot Iron; RFe, Root Iron; SMn, Shoot Manganese; RMn, Root Manganese; SCu, Shoot Copper; RCu, Root Copper; SZn, Shoot Zinc; RZn, Root Zinc.

The relative changes in root-shoot phosphorus, root-shoot potassium, root-shoot manganese, shoot magnesium, and shoot zinc content were found to be significantly associated with the relative change in shoot dry weight. Similarly, relative change in root dry weight was significantly correlated to those of root-shoot magnesium, root-shoot potassium, root-shoot zinc, root sodium, root phosphorus, root iron, and root manganese content (**Table 6**). A strong significant association was found between the shoot sodium content and root-shoot calcium, shoot iron, shoot copper, and root zinc. Similarly, root sodium was significantly related to root-shoot calcium, root magnesium, root-shoot copper, and root zinc (**Table 6**). While shoot calcium was significantly associated relative change in root magnesium, shoot iron, and shoot copper, root calcium was connected to root magnesium, shoot iron, root-shoot copper, and root zinc. Both root and shoot phosphorus were strongly associated with shoot magnesium, root-shoot potassium, root-shoot manganese, and shoot zinc content under combined drought and salinity stress (**Table 6**). In shoots, magnesium showed a significant positive correlation with shoot potassium, shoot zinc, and root-shoot manganese. However, in roots, it was associated with root-shoot copper, shoot iron, and root zinc. Similarly, root-shoot potassium was closely related to root-shoot magnesium and root iron content (**Table 6**).

### 3.10 Clustering of genotypes based on the relative changes in their nutrient content under drought, salinity, and combined drought and salinity stresses as compared to the control treatment

The generated heatmaps for all the three stress conditions clustered the genotypes on the basis of column Z-scores of the measured traits. While the red color indicates relatively low trait values, green color shows relatively high trait values. It means green patches suggest comparatively higher values for the associated traits (either dry weights or nutrient uptake) reflecting greater tolerance or adaptive responses and red patches suggest comparatively lower values for the associated traits reflecting greater sensitivity towards stress condition. The genotypes with more number of green columns especially for shoots were more tolerant to that particular stress condition.

**Figure 1:**
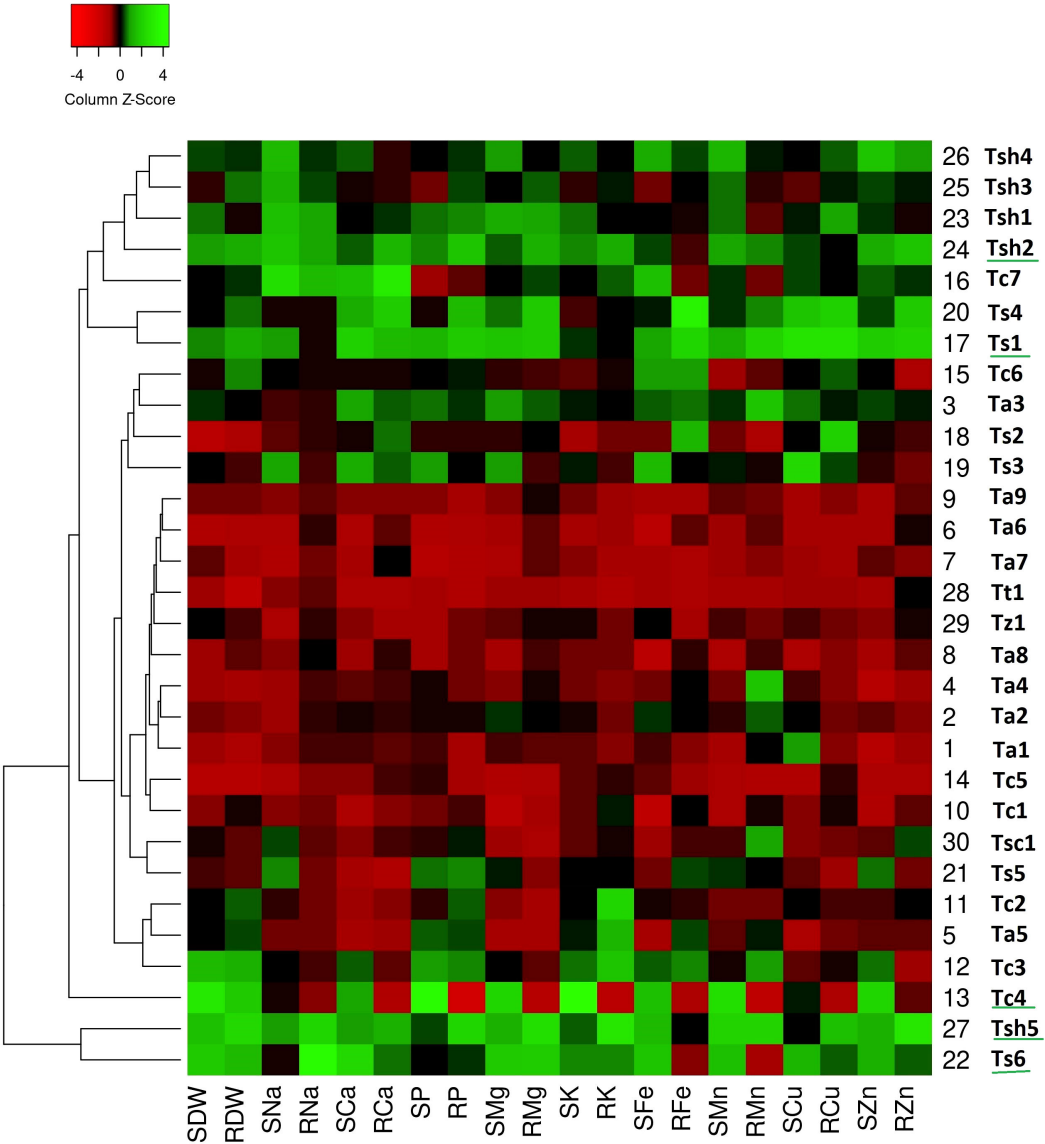
Genotype clustering based on the relative changes in their nutrient content under drought stress as compared to control treatment. [SDW - Shoot Dry Weight; RDW - Root Dry Weight; SNa - Shoot Sodium; RNa - Root Sodium; SCa - Shoot Calcium; RCa - Root Calcium; SP - Shoot Phosphorus; RP - Root Phosphorus; SMg - Shoot Magnesium; RMg - Root Magnesium; SK - Shoot Potassium; RK - Root Potassium; SFe - Shoot Iron; RFe - Root Iron; SMn - Shoot Manganese; RMn - Root Manganese; SCu - Shoot Copper; RCu -Root Copper; SZn - Shoot Zinc; RZn - Root Zinc].

**Figure 2:**
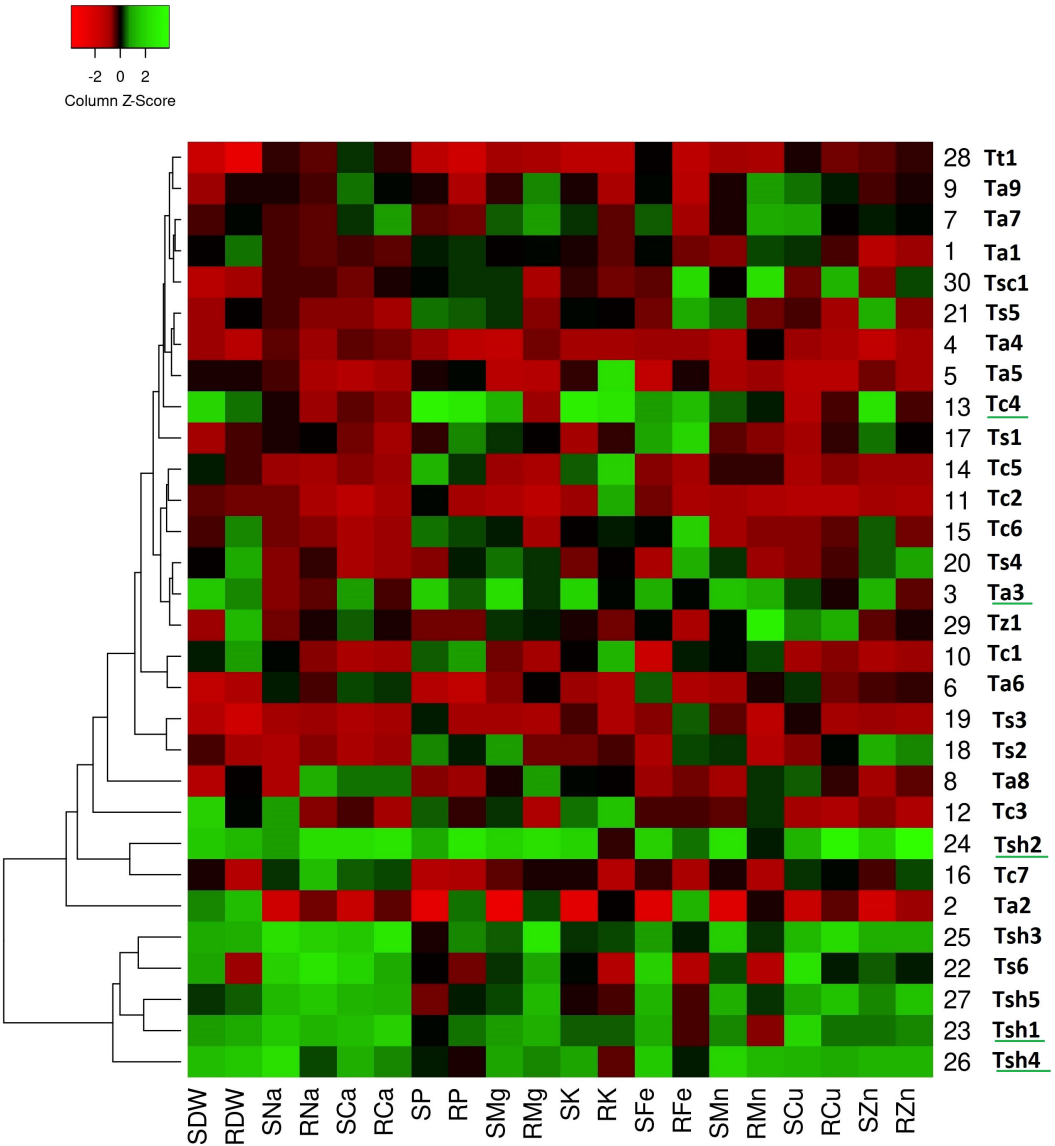
Genotype clustering based on the relative changes in their nutrient content under salinity stress as compared to control treatment. [SDW - Shoot Dry Weight; RDW - Root Dry Weight; SNa - Shoot Sodium; RNa - Root Sodium; SCa - Shoot Calcium; RCa - Root Calcium; SP - Shoot Phosphorus; RP - Root Phosphorus; SMg - Shoot Magnesium; RMg - Root Magnesium; SK - Shoot Potassium; RK - Root Potassium; SFe - Shoot Iron; RFe - Root Iron; SMn - Shoot Manganese; RMn - Root Manganese; SCu - Shoot Copper; RCu -Root Copper; SZn - Shoot Zinc; RZn - Root Zinc].

**Figure 3:**
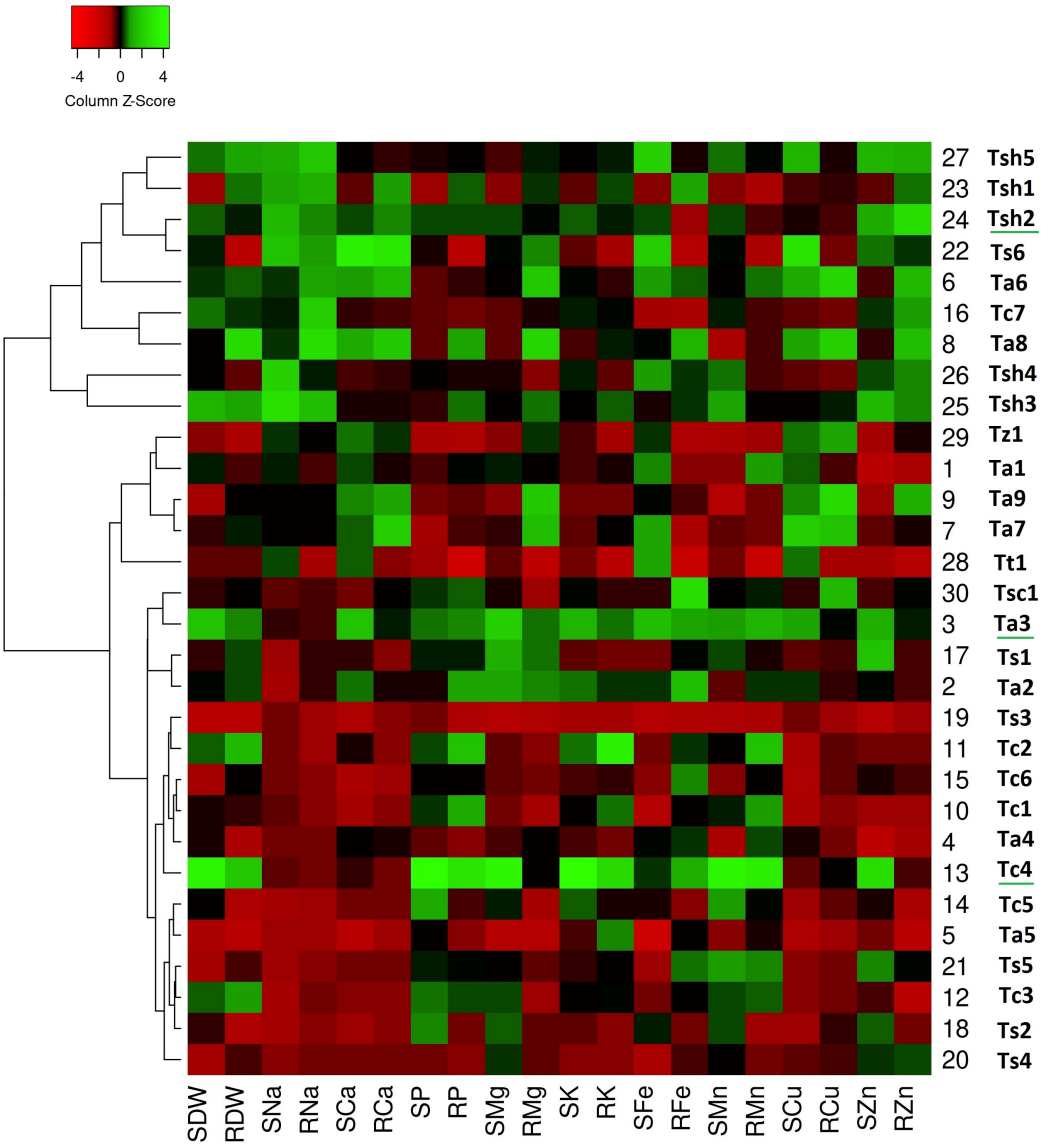
Genotype clustering based on the relative changes in their nutrient content under combined drought and salinity stress as compared to control treatment. [SDW - Shoot Dry Weight; RDW - Root Dry Weight; SNa - Shoot Sodium; RNa - Root Sodium; SCa - Shoot Calcium; RCa - Root Calcium; SP - Shoot Phosphorus; RP - Root Phosphorus; SMg - Shoot Magnesium; RMg - Root Magnesium; SK - Shoot Potassium; RK - Root Potassium; SFe - Shoot Iron; RFe - Root Iron; SMn - Shoot Manganese; RMn - Root Manganese; SCu - Shoot Copper; RCu -Root Copper; SZn - Shoot Zinc; RZn - Root Zinc].

On comparing heat maps of three stress conditions, it can be observed that in the drought stress heatmap, two clear patches of green and red colors can be seen (**Figure 1**). The green color patch include genotypes *Tc4*, *Tc7*, *Ts1*, *Ts4*, *Ts6*, *Tsh1*, *Tsh2*, *Tsh3*, *Tsh4*, and *Tsh5*, while the red color patch includes genotypes *Tt1*, *Tz1*, *Ta1*, *Ta2*, *Ta4*, *Ta6*, *Ta7*, *Ta8*, *Ta9*, *Tc1*, and *Tc5* (**Figure 1**). Similarly, in the salinity stress heatmap, the green color patch includes genotypes *Tsh1*, *Tsh2*, *Tsh3*, *Tsh4*, *Tsh5*, and *Ts6*, while the red color patch includes all the remaining genotypes except *Ta3* and *Tc4* (**Figure 2**). However, in the combined drought and salinity stress heatmap, no clear patch of red or green color revealing any clear grouping based on the studied parameters is present (**Figure 3**).

In the combined drought and salinity stress heatmap (**Figure 3**), all the genotypes were grouped into two main clusters, Cluster A and Cluster B, based on the relative changes in their nutrient content under combined stress as compared to Control. While Cluster A consists of nine genotypes, twenty-one genotypes are present in Cluster B. All five *T.* sp*haerococcum* genotypes are closely grouped in Cluster A, showing a unique high nutrient and medium biomass pattern. *T.* sp*haerococcum* genotypes are the ones with consistently higher micronutrient bioaccumulation. Similarly, except *Tc7*, all six *T. compactum* genotypes were clustered in a close subgroup of Cluster B. Among these six genotypes, except *Tc4*, all the other showed low nutrient and medium biomass.

In a similar way, except for *Ts6*, all the *T.* sp*elta* genotypes are present in Cluster B. The *T. aestivum* genotypes are present in both clusters, with the two *T. aestivum* genotypes of Cluster A showing high-low nutrient and low biomass. While all the aestivum genotypes of Cluster B showed low nutrient and low biomass, *Ta3* showed high nutrient and high biomass.

According to the drought stress heatmap, genotypes *Tc4*, *Ts1*, *Ts6*, *Tsh2*, and *Tsh5* seem to be the top performing genotypes with high biomass and high nutrient accumulation. In the salinity stress heatmap, genotypes *Ta3*, *Tc4*, *Tsh1*, *Tsh2*, and *Tsh4* seem to be the top performing genotypes with high biomass and high nutrient accumulation. In the combined drought and salinity stress heatmap, genotypes *Tc4*, *Ta3*, and *Tsh2* seem to be the top-performing genotypes with high biomass and high nutrient accumulation.

## 4 Discussion

Drought and salinity are major abiotic stresses restricting plant growth, particularly in arid and semi-arid regions (Khan et al., 2024). In these regions, they often co-occur as combined drought and salinity stress (Kumar et al., 2019; Romdhane et al., 2020; Ben Gaied et al., 2024). Through the long-term evolutionary processes, plants have developed intricate mechanisms to respond to various environmental stresses. Changes in nutrient uptake, accumulation, and distribution are one of those adaptive responses that help plants to regulate cellular homeostasis and facilitate survival under adverse conditions (Khan et al., 2023; Wang et al., 2025). Distinct germplasms of the same or different species can exhibit variable nutrient response to a particular stress depending on their growth and breeding background (Maharajan et al., 2021; Khan et al., 2022). Hence, in this study, changes in root-shoot nutrient accumulation along with the biomass were observed to understand how differently combined drought and salinity stress affect different Triticum genotypes as compared to drought and salinity stresses alone.

### 4.1 Significant genotypic variability in root and shoot nutrient accumulation under drought, salinity, and combined drought and salinity stresses

Previously, most of the studies have focused on drought and salinity stress alone as a single stress factor. However, since the last few years, there is a growing body of research on the combined effects of drought and salinity stress in wheat (Ahmed et al., 2013; Dugasa et al., 2019; Fu et al., 2024b; Shamloo-Dashtpagerdi et al., 2024). Despite this, no research has focused on the accumulation of macro- and micronutrients in wheat genotypes under combined drought and salinity stress conditions. The frequent co-existence of drought and salinity stress in field conditions may affect nutrient content in plants positively or negatively (Vasantha et al., 2017; Akhzari et al., 2022; Kucukyumuk et al., 2025). This positive or negative response of plants can direct towards their genotypic variation and hence, nutrient uptake and accumulation patterns under stress are widely used to distinguish between tolerant and susceptible genotypes in crops. In this study, accumulation of nutrients highlighted noticeable variation both at the level of genotypes and species under drought, salinity, and combined stresses.

At the genotypic level, biomass and accumulation of phosphorus, manganese, potassium, zinc, and iron in both roots and shoots, along with copper and sodium in roots and calcium in shoots, significantly contributed to the variability of the studied wheat genotypes. For instance, certain genotypes, such as *Tc4* and *Ts1*, maintained high shoot phosphorus content under drought stress compared to the Control, while *Tc4*, *Tc5*, and *Ta3* did so under salinity stress. Under combined drought and salinity stress, only *Tc4* maintained high phosphorus levels. This ability can be a reason for the high relative shoot dry weight of *Tc4* in both drought and combined stress, and of *Tc4* and *Ta3* under salinity stress. Similarly, the high relative root phosphorus observed in *Tsh5* and *Ts1* under drought and *Tc4* and *Tc2* under combined drought and salinity stress can be linked to their correspondingly high relative root dry weights in the same conditions. Under drought stress, three *T. compactum* genotypes, *Tc3*, *Tc4*, and *Tc7*; two *T.* sp*elta*, *Ts1*, and *Ts6*; one *T. aestivum*, *Ta3*, and four *T.* sp*haerococcum*, *Tsh1*, *Tsh2*, *Tsh4*, and *Tsh5* seem to be more tolerant than other genotypes of the same species, with greatest increase in biomass and nutrient accumulation when compared to Control. Under salinity stress, four *T.* sp*haerococcum*, *Tsh1*, *Tsh2*, *Tsh4*, and *Tsh5*; one *T. aestivum*, *Ta3*, and one *T. compactum*, *Tc4*, seem to be more tolerant than other genotypes of the same species, with the greatest increase in biomass and nutrient accumulation when compared to the Control. Under combined drought and salinity stress, two *T. aestivum*, *Ta3* and *Ta6*; three *T. compactum* genotypes, *Tc2*, *Tc3*, and *Tc4*; and three *T.* sp*haerococcum*, *Tsh2*, *Tsh3*, and *Tsh5*, seem to be more tolerant than other genotypes of the same species, with the greatest increase in biomass and nutrient accumulation when compared to the Control. The consistent response of *Tc4*, *Ta3*, and *Tsh5* across all three stress conditions makes them a potential candidate for breeding programs targeting multi-stressed environments.

If observed at the species level, under drought stress, *T.* sp*haerococcum* seems to be the most tolerant species with the highest increase in biomass and accumulation of most of the nutrients, especially macronutrients, when compared to the Control. Among six *T.* sp*haerococcum* genotypes, *Tsh5* was the best performing one. In contrast, *T. aestivum* turned out to be the most sensitive species, where all the genotypes, except *Ta3*, showed maximum decrease in biomass and nutrient accumulation. Surprisingly, the response of *T.* sp*haerococcum* genotypes was better than the well-known drought-tolerant bread wheat check cultivar, *Ta6* (C 306) used in this study. These findings were in accordance with the outcomes reported by Gaikwad et al. (2024) where average grain yields of the *T.* sp*haerococcum* accessions outperform the drought-tolerant cultivar C 306 under restricted irrigation, confirming its potential as a genetic resource for drought stress tolerance.

Similarly, under salinity stress as well, the genotypes of *T.* sp*haerococcum* surpassed the performance of *T. aestivum* genotypes, which also include the three well-recognized salt-tolerant cultivars, *Ta7* (K 9006), *Ta8* (KRL 210), and *Ta9* (KRL 213). Along with increased biomass, *T.* sp*haerococcum* genotypes showed better accumulation of nutrients, especially in shoots. Among the six *Triticum* sp*haerococcum* genotypes, *Tsh2* was the best performing one under salinity stress. The better performance of *Tsh5* and *Tsh2* than drought- and salt-tolerant check cultivars used in this study emphasizes that *T.* sp*haerococcum* possesses untapped allelic diversity that can be used in wheat breeding programs for improving salinity tolerance of modern cultivars (Adhikari et al., 2023; Mazumder et al., 2024). This superior performance of tolerant genotypes can be associated to nutrient translocation mechanisms, possibly involving active transport and compartmentalization of toxic ions like sodium ions and ion homeostasis. Moreover, the increase in the accumulation of nutrients such as phosphorus, potassium, and magnesium in root and shoot tissues may have contributed to osmotic adjustment and membrane stabilization of these genotypes under drought and salinity stress (Hu and Schmidhalter, 2005; Dugasa et al., 2019, 2021).

In contrast to individual drought and salinity stress, under combined drought and salinity stress, no species gave a specific pattern of higher biomass or accumulation of all the nutrients or a particular nutrient in the entire set of genotypes. Instead, some genotypes belonging to different species showed unique patterns in the accumulation of particular nutrients. For instance, while all five *T.* sp*haerococcum* genotypes showed high relative shoot sodium and root zinc content under combined drought and salinity stress as compared to the Control, only three of them showed higher shoot biomass, root phosphorus, and shoot manganese. Likewise, only six of the nine *T. aestivum* genotypes showed high shoot calcium and root magnesium accumulation under combined drought and salinity stress as compared to the Control. This highlighted a genotype-dependent response rather than a species-based response of the studied genotypes under combined stress. In line with our study, several previous studies reported that responses were significant at genotypic level and not the species level (Stavridou et al., 2019; Yadav et al., 2022). Specific combinations of physiological, biochemical, and molecular adaptations based on stress-mitigation mechanisms, such as variations in antioxidant enzyme activity, osmolyte accumulation, ion homeostasis, and stress-responsive gene expression, can be involved in maintaining the growth of some genotypes under combined drought and salinity stress (Ibrahim et al., 2019; Kayacetin, 2022; Yadav et al., 2022; Alsamadany et al., 2024; Alzahrani et al., 2025).

### 4.2 Comparing the effects of combined drought and salinity stress with drought and salinity stress on the accumulation of different nutrients

Occurrence of multiple stresses can have additive, complementary, or counteractive effects on plants depending on stress type, species, genotypes, and traits (Zandalinas and Mittler, 2022). In this study, while 2 and 3 genotypes showed an increase in shoot phosphorus content under individual drought and salinity stress, as compared to the Control, respectively, under combined drought and salinity stress, only one genotype showed an increase. Similarly, in roots, while drought and salinity stress showed an increase in 12 and 19 genotypes, respectively, only 11 genotypes showed an increase in phosphorus content under combined drought and salinity stress. Similar to phosphorus, other macronutrients, including calcium, potassium, and magnesium, along with all the micronutrients (except root copper), including iron, manganese, and zinc, showed increased accumulation in a greater number of genotypes under drought and salinity stress compared to combined drought and salinity stress. These findings highlighted that overall combined drought and salinity stress was more damaging for the studied wheat genotypes as compared to individual drought and salinity stress. A several impairment or differential regulation of physiological and biochemical pathways responsible for nutrient uptake, transport, and homeostasis under combined drought and salinity stress is suggested. The decrease in the number of genotypes capable of maintaining nutrient content under combined stress directs towards a synergistic negative effect rather than an additive one (Paul et al., 2019; Argentel-Martínez et al., 2024; Naqi et al., 2024).

Both drought and salinity exert osmotic pressure in plants by limiting water availability. However, salinity further develops ionic stress with an accumulation of toxic ions such as sodium (Na^+^) and chloride (Cl^-^). These effects are compounded under combined drought and salinity stress, decreasing the water uptake and cellular turgor as well as increasing accumulation of reactive oxygen species that consequently limit cell expansion and biomass development (Neumann, 1995; Angon et al., 2022). This was evidenced in our study by a significant reduction in SDW under combined drought and salinity stress as compared to either stress alone. The reduced sodium accumulation under combined drought and salinity stress, as compared to individual salinity stress, suggests that drought stress stimulates some other changes in the uptake and transport of sodium ions. Despite the reduced sodium levels in combined drought and salinity stress as compared to salinity stress, there are possibly some other factors, such as disturbed ionic balance and increased oxidative stress that reduced the growth under the combined stress (Zhang et al., 2023b).

As wheat plants experience both ionic and osmotic stress under combined drought and salinity stress, plants would have spent more energy on the maintenance of cellular water balance, and ion homeostasis rather than biomass accumulation (Karnik et al., 2017; Ghosh et al., 2021; Mangal et al., 2023).

Moreover, compounded energy is required due to detoxification of reactive oxygen species (ROS) and simultaneous activation of multiple defense pathways such as abscisic acid (ABA), mitogen-activated protein kinases (MAPKs), and calcium signaling and signal transduction (Choudhury et al., 2017; Ravi et al., 2023).

Salinity stress disturbs the uptake and distribution of nutrients such as potassium, calcium, magnesium, and phosphorus due to competition with sodium ions at transport sites. It also disturbs the membrane permeability in roots and interferes with xylem loading and long-distance translocation (Isayenkov, 2019; Khare et al., 2024). These disturbances are further worsened by drought due to a reduction in transpiration rates which is important for nutrient transport from roots to shoots (Hu and Schmidhalter, 2005). Accordingly, in this study, greater reductions of Mg, P, and Ca were observed in combined stress compared to individual drought and salinity stress, especially in shoots. Additionally, shoots were more affected than roots under combined stress in most of the nutrients. Although some genotypes such as *Tc4*, *Ta3*, and *Tsh2* showed better nutrient accumulation, and biomass under combined drought and salinity stress, most of them showed significant reductions, highlighting the greater negative effect of combined drought and salinity stress.

Roots are the main organs that sense water availability in soil which transmit the required hormonal and electrical signals to the shoots for coordinating a proper response towards stress. The abscisic acid and its receptor-mediated pathways that are involved in wheat signaling under drought regulate closure of stomata, osmolyte accumulation and antioxidant responses to prevent water loss (Guizani et al., 2023; Zhang et al., 2023a, 2025). Different macroelements such as potassium and calcium, and microelements participate in the stabilization of ion homeostasis and osmotic adjustment via these pathways (Vuković et al., 2022; Guizani et al., 2023; Fu et al., 2024a). Under salinity stress, several macroelements such as calcium, potassium, and sodium, and micronutrients such as Fe, Zn, Cu, and Mn are involved in signaling. Ca^2+^ ions act as ubiquitous secondary messenger and their levels rapidly increase under high salt to transfer stress signals and stimulate adaptive responses (Dugasa et al., 2021; Lindberg and Premkumar, 2023). Potassium and sodium ions compete during uptake and signaling pathways such as SOS pathway and Na^+^/H^+^ antiporters act to exclude or compartmentalize sodium ions in different wheat tissues (Wu et al., 2015; Lindberg and Premkumar, 2023). While drought induces the abscisic acid accumulation, facilitating the closure of stomata to prevent water loss, salinity induces ion toxicity (Angon et al., 2022). This signaling can become more complex under combined drought and salinity stress. The simultaneous presence of both stresses disturbs the coordinated root-shoot signaling, leading to abrupt physiological responses such as closing of stomata despite continuous influx of sodium ions (Pierik and Testerink, 2014; Hussain et al., 2021; Angon et al., 2022; Pandey et al., 2023). Accordingly, in this study, accumulation of nutrients such as potassium, calcium, and magnesium was much reduced in shoots as compared to roots, highlighting the possible breakdown in coordinated interaction between roots and shoots, and interference in signaling between them (Hussain et al., 2021). A deeper research is required related to molecular and physiological mechanisms underlying root-to-shoot signaling under combined drought and salinity stress.

## 5 Conclusion

This study explores genotypic variation in nutrient uptake and biomass accumulation among 30 hexaploid wheat genotypes from seven species, evaluated under drought, salinity, and combined drought and salinity stress. Moreover, the study also attempted to understand the mechanisms underlying the variation in nutrient accumulation. Significant species and genotypic level differences were observed in the nutrient accumulation of these genotypes under these stresses, with a more complex and genotype-dependent response noticed under combined drought and salinity stress. Though individual drought and salinity stress resulted in considerable reductions in both dry weights and nutrient accumulation, their simultaneous existence had a more damaging and synergistic negative effect, specifically on dry weights and macronutrients such as phosphorus, potassium, magnesium, and calcium. Among the studied genotypes, *Tc4* (PI 164160, Kanak, India) was the best-performing genotype across all three stress conditions, followed by *Ta3* (CItr 17028, CAR 1101, Chile) and *Tsh2* (PI 42013, India) in terms of improved nutrient accumulation and biomass, and can be used for future genetic improvement.

Superior adaptability of *T.* sp*haerococcum* genotypes under both drought and salinity stress suggested that this species is a reservoir of valuable alleles that can be used to improve wheat resilience towards abiotic stresses. The underperformance of *T. aestivum* genotypes in the study, including the well-established drought and salt-tolerant check cultivars, emphasizes the necessity of exploring a greater number of underutilized wheat species in breeding programs. The contrasting genotype-dependent responses under combined stress, contrary to the uniform species level responses under individual drought and salinity stress, indicated that adaptation under combined stress involves complex and genotype-dependent mechanisms. Hence, a greater number of wheat genotypes must be evaluated under realistic multi-stress environments rather than individual stresses. The greater decreases in nutrient content under combined drought and salinity stress, particularly in shoots may indicate a disruption in root-shoot communication. This also emphasized the need for further transcriptomic or proteomic studies to understand the underlying molecular mechanisms for differential nutrient uptake under nutrient stress. Moreover, root morphology and architecture should be studied in detail to identify physical barriers to nutrient absorption under combined stress.

The results obtained in this study identified potential genotypes and species that can be used for breeding programs targeting individual drought, salinity, and combined drought and salinity stress. In addition, the study emphasized that combined drought and salinity stress is not just a sum of drought and salinity stress, but it involves a complex interaction of multiple stress pathways, leading to a greater physiological and biochemical disruption. Hence, it should be thoroughly explored as a separate stress condition to identify a greater number of genetic resources that are tolerant to this deadly stress combination. The results provided valuable insights emphasizing the relevance of nutrient profiling as a critical component of breeding frameworks for climate-resilient wheat.

## Data Availability

The original contributions presented in the study are included in the article/**Supplementary Material**, further inquiries can be directed to the corresponding author/s.
