# Supplementary material for: Nutrient uptake under combined drought and salinity stress in hexaploid wheat species
Source: Front Plant Sci. 2025 Nov 10;16:1682258. doi: 10.3389/fpls.2025.1682258 (PMC12641000; doi:10.3389/fpls.2025.1682258)
Supplement: Supplementary file 1 [file SupplementaryFile1.zip › Revised Supplementary Files/Supplementary_Material.docx]

Supplementary Material

# Supplementary Figures and Tables

## Supplementary Figures

**Supplementary Figure S1-S20.** Variation in the shoot and root biomass, sodium (Na), calcium (Ca), potassium (K), magnesium (Mg), phosphorus (P), iron (Fe), manganese (Mn), copper (Cu), and zinc (Zn) uptake, respectively in 30 hexaploid wheat genotypes under Control (C), drought (D), salinity (S) and combined drought and salinity (D+S) treatments. While x-axis presents the genotypes, y axis presents the trait.

## Supplementary Tables

**Supplementary Table S1**. The shoot and root biomass, sodium (Na) and calcium (Ca) uptake in 30 hexaploid wheat genotypes under Control (C), drought (D), salinity (S) and combined drought and salinity (D+S) treatments. Biomass is presented in ‘g’, nutrient uptake is presented as µg/plant.

| **Genotype Code** | **Shoot Dry Weight** | | | | **Root Dry Weight** | | | | **Accum Shoot Na** | | | | **Accum Root Na** | | | | **Accum Shoot Ca** | | | | **Accum Root Ca** | | | |
| --- | --- | --- | --- | --- | --- | --- | --- | --- | --- | --- | --- | --- | --- | --- | --- | --- | --- | --- | --- | --- | --- | --- | --- | --- |
|  | **C** | **D** | **S** | **D+S** | **C** | **D** | **S** | **D+S** | **C** | **D** | **S** | **D+S** | **C** | **D** | **S** | **D+S** | **C** | **D** | **S** | **D+S** | **C** | **D** | **S** | **D+S** |
| **Ta1** | **0.035** | **0.024** | **0.027** | **0.019** | **0.008** | **0.006** | **0.010** | **0.007** | **6.0** | **4.5** | **307.7** | **166.2** | **9.6** | **6.1** | **100.1** | **85.3** | **239.0** | **138.7** | **86.5** | **105.9** | **122.9** | **84.5** | **101.3** | **75.4** |
| **Ta2** | **0.036** | **0.026** | **0.033** | **0.020** | **0.008** | **0.008** | **0.012** | **0.010** | **9.0** | **5.5** | **22.7** | **88.1** | **12.3** | **9.3** | **123.5** | **132.9** | **244.3** | **159.1** | **13.3** | **119.6** | **98.9** | **81.7** | **83.1** | **69.6** |
| **Ta3** | **0.032** | **0.030** | **0.033** | **0.024** | **0.008** | **0.011** | **0.011** | **0.010** | **4.8** | **4.6** | **199.1** | **95.2** | **12.0** | **9.2** | **128.8** | **116.1** | **169.6** | **165.0** | **108.4** | **116.3** | **79.3** | **111.3** | **81.8** | **73.8** |
| **Ta4** | **0.045** | **0.031** | **0.029** | **0.023** | **0.012** | **0.010** | **0.010** | **0.009** | **8.1** | **5.3** | **397.6** | **122.5** | **15.0** | **8.9** | **90.8** | **92.1** | **287.6** | **151.8** | **97.7** | **103.1** | **100.7** | **75.7** | **64.7** | **64.3** |
| **Ta5** | **0.043** | **0.037** | **0.032** | **0.016** | **0.008** | **0.012** | **0.009** | **0.005** | **9.5** | **7.4** | **500.4** | **99.2** | **25.2** | **6.7** | **101.2** | **52.4** | **272.6** | **108.8** | **45.9** | **26.4** | **111.3** | **50.9** | **7.8** | **7.9** |
| **Ta6** | **0.052** | **0.030** | **0.028** | **0.030** | **0.012** | **0.010** | **0.011** | **0.014** | **6.3** | **2.5** | **428.6** | **183.2** | **6.5** | **5.0** | **79.0** | **178.3** | **222.8** | **82.6** | **123.3** | **117.3** | **34.6** | **24.1** | **68.4** | **72.1** |
| **Ta7** | **0.042** | **0.032** | **0.030** | **0.021** | **0.011** | **0.009** | **0.013** | **0.012** | **4.2** | **1.6** | **219.4** | **98.3** | **11.5** | **3.0** | **128.9** | **166.2** | **217.9** | **89.3** | **110.5** | **102.1** | **34.0** | **32.2** | **96.8** | **85.2** |
| **Ta8** | **0.039** | **0.026** | **0.022** | **0.020** | **0.009** | **0.009** | **0.010** | **0.015** | **2.9** | **2.0** | **80.6** | **86.4** | **3.8** | **4.8** | **122.5** | **192.3** | **169.3** | **72.7** | **100.2** | **97.2** | **31.1** | **25.0** | **79.7** | **75.4** |
| **Ta9** | **0.050** | **0.036** | **0.032** | **0.019** | **0.012** | **0.012** | **0.014** | **0.013** | **4.3** | **2.9** | **246.9** | **104.6** | **10.7** | **5.2** | **134.7** | **157.0** | **186.0** | **85.8** | **110.9** | **94.7** | **50.5** | **27.9** | **84.6** | **82.3** |
| **Tc1** | **0.048** | **0.034** | **0.038** | **0.024** | **0.008** | **0.010** | **0.011** | **0.008** | **6.0** | **4.2** | **388.9** | **93.3** | **19.4** | **6.5** | **148.9** | **92.4** | **256.4** | **98.0** | **50.9** | **47.5** | **88.4** | **49.3** | **8.8** | **9.6** |
| **Tc2** | **0.038** | **0.033** | **0.026** | **0.023** | **0.006** | **0.010** | **0.006** | **0.009** | **7.8** | **8.8** | **348.8** | **116.6** | **27.9** | **6.6** | **79.9** | **91.1** | **255.8** | **112.9** | **29.1** | **85.7** | **90.2** | **48.3** | **6.0** | **13.9** |
| **Tc3** | **0.027** | **0.033** | **0.029** | **0.016** | **0.006** | **0.012** | **0.007** | **0.008** | **6.9** | **9.4** | **600.8** | **68.1** | **15.3** | **9.3** | **114.7** | **80.1** | **129.4** | **107.9** | **47.8** | **29.8** | **75.5** | **53.4** | **5.1** | **9.3** |
| **Tc4** | **0.025** | **0.040** | **0.028** | **0.026** | **0.006** | **0.014** | **0.008** | **0.010** | **7.2** | **8.6** | **415.9** | **124.8** | **18.6** | **0.3** | **122.4** | **109.4** | **135.9** | **134.0** | **44.5** | **41.8** | **54.1** | **10.4** | **20.7** | **14.3** |
| **Tc5** | **0.050** | **0.028** | **0.040** | **0.026** | **0.015** | **0.009** | **0.015** | **0.010** | **13.7** | **5.8** | **541.0** | **124.2** | **46.6** | **7.0** | **207.4** | **117.7** | **183.7** | **85.4** | **54.3** | **44.4** | **74.4** | **54.4** | **22.9** | **16.6** |
| **Tc6** | **0.035** | **0.030** | **0.025** | **0.014** | **0.004** | **0.007** | **0.006** | **0.005** | **5.6** | **7.4** | **255.5** | **84.2** | **9.5** | **9.3** | **76.2** | **47.9** | **124.1** | **80.6** | **24.9** | **20.1** | **61.3** | **54.8** | **10.8** | **5.8** |
| **Tc7** | **0.054** | **0.049** | **0.040** | **0.034** | **0.012** | **0.018** | **0.011** | **0.014** | **4.4** | **15.0** | **305.5** | **121.0** | **4.5** | **15.8** | **168.6** | **187.8** | **226.0** | **263.1** | **131.9** | **67.4** | **39.0** | **120.6** | **85.0** | **18.6** |
| **Ts1** | **0.044** | **0.047** | **0.028** | **0.021** | **0.007** | **0.014** | **0.008** | **0.009** | **6.7** | **13.8** | **393.3** | **77.6** | **6.7** | **7.3** | **107.3** | **76.7** | **108.5** | **140.3** | **35.2** | **32.7** | **46.2** | **91.2** | **7.2** | **7.4** |
| **Ts2** | **0.046** | **0.024** | **0.033** | **0.023** | **0.010** | **0.008** | **0.009** | **0.007** | **9.1** | **8.5** | **258.6** | **81.6** | **13.5** | **12.0** | **113.6** | **58.8** | **161.8** | **102.9** | **32.8** | **32.6** | **69.6** | **103.1** | **15.6** | **12.3** |
| **Ts3** | **0.052** | **0.046** | **0.029** | **0.017** | **0.010** | **0.011** | **0.007** | **0.006** | **6.0** | **12.7** | **206.6** | **84.0** | **17.3** | **10.5** | **121.4** | **46.4** | **165.7** | **166.3** | **32.2** | **23.5** | **57.4** | **78.8** | **8.8** | **11.4** |
| **Ts4** | **0.049** | **0.044** | **0.038** | **0.019** | **0.008** | **0.013** | **0.011** | **0.008** | **6.2** | **7.3** | **254.5** | **73.6** | **8.7** | **8.7** | **119.5** | **57.0** | **113.8** | **115.1** | **25.5** | **28.0** | **36.8** | **83.3** | **8.6** | **10.5** |
| **Ts5** | **0.037** | **0.029** | **0.024** | **0.014** | **0.007** | **0.007** | **0.008** | **0.006** | **6.3** | **12.2** | **325.0** | **63.7** | **13.8** | **3.3** | **99.1** | **60.4** | **152.0** | **63.3** | **44.8** | **36.5** | **57.8** | **13.0** | **8.2** | **13.8** |
| **Ts6** | **0.039** | **0.051** | **0.035** | **0.021** | **0.008** | **0.016** | **0.007** | **0.005** | **2.5** | **2.9** | **303.3** | **127.6** | **2.1** | **15.2** | **115.1** | **55.8** | **102.8** | **140.8** | **96.5** | **105.9** | **26.3** | **37.5** | **85.4** | **91.8** |
| **Tsh1** | **0.030** | **0.031** | **0.027** | **0.012** | **0.007** | **0.009** | **0.009** | **0.008** | **2.5** | **6.7** | **292.6** | **102.1** | **3.2** | **9.4** | **108.3** | **103.8** | **162.9** | **117.1** | **128.1** | **42.7** | **17.0** | **20.6** | **81.3** | **25.7** |
| **Tsh2** | **0.027** | **0.030** | **0.029** | **0.017** | **0.006** | **0.011** | **0.008** | **0.006** | **2.4** | **6.5** | **207.1** | **116.1** | **2.8** | **8.1** | **141.7** | **73.1** | **118.8** | **100.7** | **121.3** | **52.6** | **15.6** | **29.9** | **94.0** | **22.3** |
| **Tsh3** | **0.030** | **0.025** | **0.028** | **0.022** | **0.006** | **0.011** | **0.009** | **0.008** | **2.1** | **4.8** | **285.5** | **142.6** | **2.9** | **5.5** | **132.5** | **106.6** | **145.1** | **93.7** | **124.6** | **48.7** | **17.6** | **15.3** | **107.7** | **10.9** |
| **Tsh4** | **0.031** | **0.030** | **0.031** | **0.016** | **0.007** | **0.010** | **0.010** | **0.006** | **1.7** | **4.3** | **235.7** | **102.6** | **4.8** | **8.4** | **109.3** | **79.9** | **135.8** | **115.2** | **99.4** | **38.6** | **28.0** | **22.6** | **76.6** | **15.8** |
| **Tsh5** | **0.032** | **0.040** | **0.025** | **0.020** | **0.006** | **0.016** | **0.008** | **0.008** | **2.1** | **4.3** | **212.1** | **90.1** | **2.7** | **12.9** | **105.6** | **105.3** | **137.2** | **130.9** | **104.6** | **48.8** | **21.5** | **39.6** | **73.2** | **11.7** |
| **Tt1** | **0.039** | **0.027** | **0.019** | **0.018** | **0.015** | **0.008** | **0.008** | **0.014** | **4.6** | **3.4** | **251.5** | **146.7** | **7.1** | **3.4** | **79.0** | **4.4** | **224.8** | **81.7** | **115.7** | **103.8** | **65.9** | **21.2** | **74.9** | **7.3** |
| **Tz1** | **0.047** | **0.042** | **0.030** | **0.020** | **0.013** | **0.015** | **0.018** | **0.010** | **5.6** | **2.6** | **251.3** | **170.7** | **11.6** | **9.7** | **171.5** | **159.5** | **207.8** | **98.3** | **119.3** | **99.0** | **68.2** | **27.6** | **88.4** | **69.2** |
| **Tsc1** | **0.046** | **0.039** | **0.026** | **0.022** | **0.013** | **0.014** | **0.012** | **0.013** | **3.5** | **5.8** | **180.3** | **56.9** | **15.0** | **6.2** | **186.5** | **133.3** | **139.4** | **65.2** | **44.3** | **34.7** | **24.8** | **19.7** | **30.0** | **19.4** |

**Supplementary Table S2**. The shoot and root potassium (K), magnesium (Mg) and phosphorus (P) uptake in 30 hexaploid wheat genotypes under Control (C), drought (D), salinity (S) and combined drought and salinity (D+S) treatments. Nutrient uptake is presented as µg/plant.

| **Genotype Code** | **Accum Shoot K** | | | | **Accum Root K** | | | | **Accum Shoot Mg** | | | | **Accum Root Mg** | | | | **Accum Shoot P** | | | | **Accum Root P** | | | |
| --- | --- | --- | --- | --- | --- | --- | --- | --- | --- | --- | --- | --- | --- | --- | --- | --- | --- | --- | --- | --- | --- | --- | --- | --- |
|  | **C** | **D** | **S** | **D+S** | **C** | **D** | **S** | **D+S** | **C** | **D** | **S** | **D+S** | **C** | **D** | **S** | **D+S** | **C** | **D** | **S** | **D+S** | **C** | **D** | **S** | **D+S** |
| **Ta1** | **2002.4** | **1207.0** | **908.7** | **677.0** | **179.2** | **151.4** | **53.4** | **54.1** | **102.9** | **56.3** | **48.3** | **43.4** | **28.7** | **14.7** | **16.7** | **11.8** | **243.2** | **150.2** | **185.8** | **112.2** | **41.1** | **24.6** | **52.8** | **37.8** |
| **Ta2** | **2124.1** | **1543.9** | **55.6** | **1113.8** | **221.9** | **202.6** | **81.0** | **91.9** | **106.5** | **73.1** | **5.3** | **54.9** | **24.0** | **16.8** | **16.9** | **14.8** | **319.4** | **215.8** | **19.4** | **161.7** | **47.8** | **39.6** | **66.9** | **60.7** |
| **Ta3** | **1891.4** | **1613.3** | **1557.9** | **1227.6** | **240.9** | **310.9** | **92.8** | **116.9** | **80.5** | **66.8** | **65.8** | **51.9** | **19.2** | **18.4** | **13.0** | **11.6** | **225.8** | **196.1** | **268.5** | **152.9** | **50.8** | **51.2** | **68.8** | **60.5** |
| **Ta4** | **2821.9** | **1662.7** | **889.6** | **970.3** | **313.4** | **266.4** | **68.6** | **65.9** | **124.4** | **58.5** | **32.6** | **44.3** | **29.2** | **19.0** | **11.1** | **12.1** | **329.1** | **219.6** | **174.3** | **141.9** | **84.8** | **57.5** | **57.1** | **52.2** |
| **Ta5** | **1622.6** | **1390.3** | **686.0** | **553.3** | **82.1** | **194.0** | **68.4** | **42.4** | **120.4** | **49.9** | **36.6** | **28.6** | **40.7** | **13.0** | **7.9** | **4.0** | **213.4** | **180.2** | **142.8** | **111.6** | **39.0** | **39.8** | **47.2** | **23.8** |
| **Ta6** | **3314.4** | **1468.8** | **1118.2** | **1379.8** | **405.7** | **296.9** | **71.4** | **111.9** | **120.0** | **52.0** | **45.1** | **47.9** | **23.0** | **11.6** | **12.7** | **20.1** | **555.1** | **215.5** | **232.3** | **232.2** | **86.7** | **47.6** | **54.5** | **69.5** |
| **Ta7** | **2937.5** | **1589.0** | **1487.9** | **918.7** | **377.3** | **251.5** | **108.0** | **127.0** | **110.2** | **44.2** | **60.7** | **41.1** | **19.1** | **10.1** | **16.0** | **15.8** | **463.1** | **168.6** | **283.1** | **155.0** | **73.2** | **37.6** | **69.7** | **54.3** |
| **Ta8** | **2626.2** | **1538.3** | **1263.3** | **904.3** | **319.1** | **286.6** | **119.6** | **119.7** | **111.9** | **47.4** | **51.8** | **38.2** | **22.1** | **12.9** | **18.6** | **23.1** | **380.0** | **176.0** | **207.0** | **159.4** | **50.0** | **35.0** | **44.4** | **64.8** |
| **Ta9** | **3194.3** | **1764.0** | **1447.2** | **936.6** | **457.8** | **337.7** | **93.2** | **86.6** | **118.4** | **56.0** | **53.0** | **38.3** | **21.7** | **14.3** | **17.7** | **20.2** | **382.3** | **196.8** | **261.6** | **153.7** | **79.1** | **46.7** | **60.2** | **55.4** |
| **Tc1** | **2150.4** | **1334.8** | **981.9** | **849.9** | **116.6** | **172.3** | **69.5** | **58.3** | **139.3** | **43.9** | **55.4** | **46.2** | **35.6** | **10.9** | **10.3** | **6.8** | **244.4** | **132.7** | **205.0** | **143.3** | **32.8** | **25.2** | **48.0** | **44.0** |
| **Tc2** | **1664.7** | **1334.5** | **541.1** | **877.5** | **54.6** | **163.3** | **30.5** | **64.0** | **110.6** | **50.8** | **35.0** | **37.6** | **27.7** | **8.8** | **3.1** | **6.9** | **205.3** | **129.5** | **148.7** | **125.0** | **22.3** | **23.4** | **18.8** | **34.5** |
| **Tc3** | **1153.5** | **1183.3** | **654.6** | **462.0** | **75.9** | **194.9** | **49.9** | **27.9** | **67.8** | **42.5** | **36.2** | **30.8** | **20.9** | **10.5** | **4.4** | **4.6** | **138.5** | **128.6** | **117.9** | **92.2** | **27.1** | **31.1** | **29.0** | **28.4** |
| **Tc4** | **647.7** | **1603.6** | **652.8** | **774.1** | **59.2** | **16.7** | **52.2** | **53.9** | **53.7** | **61.1** | **35.7** | **45.4** | **20.3** | **3.1** | **6.6** | **8.4** | **107.4** | **184.8** | **165.7** | **158.9** | **26.7** | **6.0** | **59.3** | **55.4** |
| **Tc5** | **1493.4** | **935.0** | **838.3** | **765.8** | **159.6** | **180.3** | **118.5** | **51.2** | **101.2** | **36.0** | **37.9** | **42.2** | **41.7** | **9.4** | **9.7** | **7.5** | **203.8** | **131.0** | **206.6** | **156.6** | **63.0** | **38.1** | **81.3** | **46.4** |
| **Tc6** | **1604.0** | **1006.1** | **744.8** | **541.7** | **94.6** | **119.1** | **38.0** | **26.8** | **67.9** | **39.5** | **34.1** | **23.0** | **17.4** | **10.2** | **4.9** | **4.6** | **168.2** | **117.8** | **145.4** | **89.8** | **21.8** | **20.6** | **28.4** | **19.1** |
| **Tc7** | **3645.8** | **2926.3** | **1640.3** | **1619.9** | **380.9** | **656.2** | **59.0** | **134.2** | **126.4** | **83.7** | **51.8** | **42.8** | **31.8** | **28.8** | **16.6** | **12.4** | **572.6** | **284.7** | **242.8** | **243.9** | **82.7** | **59.7** | **65.7** | **55.2** |
| **Ts1** | **1723.8** | **1567.0** | **526.4** | **541.1** | **175.3** | **236.6** | **59.2** | **37.5** | **53.5** | **54.2** | **28.6** | **29.6** | **13.7** | **21.1** | **7.5** | **8.1** | **222.5** | **240.6** | **147.0** | **123.9** | **31.5** | **46.3** | **45.4** | **31.3** |
| **Ts2** | **1792.2** | **815.6** | **658.8** | **567.5** | **210.3** | **184.9** | **66.8** | **36.7** | **85.1** | **47.5** | **50.8** | **40.2** | **22.1** | **17.3** | **8.2** | **6.4** | **212.8** | **137.4** | **191.0** | **147.6** | **38.6** | **30.5** | **47.3** | **25.6** |
| **Ts3** | **2006.1** | **1779.9** | **821.5** | **476.8** | **195.2** | **204.2** | **34.8** | **22.7** | **85.2** | **70.6** | **30.9** | **20.2** | **29.2** | **16.7** | **6.7** | **3.6** | **237.4** | **224.4** | **182.2** | **93.3** | **38.6** | **34.2** | **32.8** | **17.0** |
| **Ts4** | **2152.0** | **1375.0** | **786.6** | **598.1** | **182.4** | **251.3** | **69.2** | **31.0** | **65.9** | **50.9** | **37.4** | **29.1** | **13.3** | **20.4** | **8.8** | **4.1** | **310.9** | **208.9** | **174.3** | **120.7** | **33.4** | **45.2** | **41.1** | **19.7** |
| **Ts5** | **1608.5** | **1333.9** | **788.7** | **566.9** | **125.1** | **172.1** | **47.0** | **41.2** | **74.4** | **49.9** | **39.7** | **29.8** | **20.2** | **8.5** | **7.1** | **5.8** | **176.6** | **158.0** | **152.6** | **98.2** | **31.5** | **35.9** | **42.7** | **29.1** |
| **Ts6** | **2399.4** | **2562.0** | **1181.2** | **771.7** | **265.2** | **508.5** | **40.7** | **29.1** | **88.8** | **91.1** | **46.0** | **36.5** | **12.7** | **20.0** | **11.4** | **8.1** | **306.8** | **227.2** | **218.4** | **153.2** | **45.0** | **45.6** | **43.3** | **15.7** |
| **Tsh1** | **1877.3** | **1929.8** | **1042.4** | **596.0** | **196.2** | **276.7** | **90.0** | **84.7** | **83.8** | **73.4** | **50.6** | **25.6** | **14.1** | **16.2** | **13.2** | **7.0** | **239.5** | **213.2** | **176.3** | **87.2** | **38.1** | **43.7** | **52.9** | **42.6** |
| **Tsh2** | **1610.8** | **1770.8** | **1336.9** | **827.5** | **155.9** | **334.6** | **51.5** | **59.6** | **75.2** | **57.4** | **58.9** | **33.6** | **10.1** | **12.8** | **14.1** | **4.5** | **181.6** | **165.9** | **177.8** | **111.6** | **26.9** | **39.0** | **59.2** | **27.9** |
| **Tsh3** | **2275.5** | **1567.7** | **1181.6** | **900.6** | **168.8** | **254.1** | **76.0** | **79.3** | **76.5** | **47.5** | **43.1** | **30.9** | **10.0** | **9.8** | **15.1** | **6.2** | **282.2** | **156.5** | **195.0** | **135.1** | **31.7** | **32.3** | **45.7** | **35.9** |
| **Tsh4** | **1921.7** | **1885.6** | **1211.2** | **871.2** | **210.8** | **272.6** | **61.7** | **46.6** | **89.7** | **73.4** | **55.3** | **35.1** | **16.6** | **13.0** | **13.2** | **4.2** | **253.4** | **181.7** | **188.8** | **131.4** | **36.5** | **36.1** | **40.8** | **29.8** |
| **Tsh5** | **2250.2** | **2281.0** | **994.9** | **910.5** | **147.4** | **509.3** | **47.2** | **55.5** | **80.7** | **73.0** | **43.8** | **29.1** | **11.6** | **21.5** | **11.9** | **5.5** | **268.2** | **217.8** | **156.6** | **134.4** | **33.4** | **53.9** | **41.2** | **29.9** |
| **Tt1** | **2297.7** | **1054.1** | **509.8** | **686.7** | **510.5** | **222.5** | **55.2** | **7.8** | **125.5** | **54.9** | **44.4** | **42.2** | **53.8** | **18.1** | **12.5** | **1.4** | **388.5** | **177.3** | **141.4** | **134.9** | **99.2** | **49.7** | **46.9** | **5.9** |
| **Tz1** | **2910.0** | **2123.9** | **1317.6** | **949.2** | **492.4** | **426.3** | **138.5** | **56.0** | **119.1** | **62.4** | **61.5** | **37.4** | **36.6** | **23.4** | **23.1** | **18.7** | **540.5** | **254.4** | **310.9** | **161.4** | **88.8** | **60.8** | **86.1** | **36.1** |
| **Tsc1** | **2186.1** | **1377.5** | **930.0** | **918.3** | **263.9** | **318.6** | **74.3** | **77.0** | **102.8** | **45.9** | **54.1** | **39.5** | **51.7** | **14.1** | **13.0** | **10.8** | **315.4** | **197.7** | **232.3** | **185.0** | **51.8** | **49.5** | **66.3** | **58.0** |

**Supplementary Table S3**. The shoot and root iron (Fe), and manganese (Mn) uptake in 30 hexaploid wheat genotypes under Control (C), drought (D), salinity (S) and combined drought and salinity (D+S) treatments. Nutrient uptake is presented as µg/plant.

| **Genotype Code** | **Accum Shoot Fe** | | | | **Accum Root Fe** | | | | **Accum Shoot Mn** | | | | **Accum Root Mn** | | | |
| --- | --- | --- | --- | --- | --- | --- | --- | --- | --- | --- | --- | --- | --- | --- | --- | --- |
|  | **C** | **D** | **S** | **D+S** | **C** | **D** | **S** | **D+S** | **C** | **D** | **S** | **D+S** | **C** | **D** | **S** | **D+S** |
| **Ta1** | **5.06** | **3.65** | **3.95** | **3.56** | **23.98** | **7.87** | **21.12** | **8.12** | **1.61** | **0.80** | **0.78** | **0.65** | **0.42** | **0.27** | **0.30** | **0.24** |
| **Ta2** | **4.24** | **3.94** | **0.85** | **2.65** | **15.51** | **8.93** | **30.60** | **15.41** | **1.65** | **1.19** | **0.08** | **0.77** | **0.72** | **0.58** | **0.40** | **0.33** |
| **Ta3** | **4.01** | **4.02** | **4.04** | **3.45** | **20.80** | **17.92** | **26.15** | **16.94** | **1.34** | **1.22** | **1.37** | **1.03** | **0.44** | **0.49** | **0.40** | **0.30** |
| **Ta4** | **5.75** | **3.70** | **3.24** | **3.25** | **20.88** | **13.37** | **14.98** | **12.84** | **2.48** | **1.52** | **0.91** | **0.83** | **0.55** | **0.62** | **0.31** | **0.26** |
| **Ta5** | **6.13** | **3.49** | **2.39** | **1.20** | **12.36** | **9.44** | **13.98** | **6.48** | **1.40** | **0.91** | **0.58** | **0.56** | **0.41** | **0.28** | **0.14** | **0.15** |
| **Ta6** | **5.20** | **2.30** | **4.58** | **3.73** | **30.00** | **11.96** | **16.27** | **20.59** | **2.43** | **1.32** | **1.06** | **1.37** | **0.88** | **0.45** | **0.48** | **0.46** |
| **Ta7** | **3.79** | **2.06** | **3.34** | **2.83** | **45.39** | **8.50** | **29.47** | **9.81** | **2.48** | **1.32** | **1.49** | **1.13** | **1.06** | **0.48** | **0.94** | **0.27** |
| **Ta8** | **4.79** | **2.13** | **2.70** | **2.79** | **25.11** | **12.40** | **21.90** | **23.42** | **2.68** | **1.25** | **1.18** | **0.91** | **1.23** | **0.67** | **0.81** | **0.37** |
| **Ta9** | **4.61** | **2.57** | **3.61** | **2.66** | **53.25** | **10.80** | **21.07** | **23.40** | **2.88** | **1.87** | **1.73** | **0.82** | **1.25** | **0.61** | **1.02** | **0.31** |
| **Tc1** | **8.37** | **3.15** | **2.91** | **2.57** | **11.16** | **6.76** | **14.94** | **5.80** | **1.88** | **0.89** | **1.25** | **1.14** | **0.63** | **0.38** | **0.45** | **0.36** |
| **Tc2** | **4.34** | **3.49** | **2.67** | **1.95** | **12.25** | **6.09** | **7.41** | **7.76** | **1.61** | **1.02** | **0.69** | **0.91** | **0.49** | **0.23** | **0.13** | **0.37** |
| **Tc3** | **3.56** | **3.52** | **2.42** | **1.58** | **8.52** | **7.92** | **8.24** | **4.51** | **1.09** | **0.85** | **0.57** | **0.72** | **0.32** | **0.29** | **0.21** | **0.16** |
| **Tc4** | **2.98** | **3.77** | **2.84** | **1.89** | **6.35** | **1.08** | **13.27** | **5.50** | **0.84** | **1.40** | **0.64** | **1.16** | **0.35** | **0.08** | **0.22** | **0.39** |
| **Tc5** | **4.04** | **2.78** | **2.42** | **2.17** | **20.34** | **5.14** | **14.15** | **6.85** | **1.40** | **0.66** | **0.83** | **1.05** | **0.95** | **0.32** | **0.50** | **0.39** |
| **Tc6** | **2.97** | **3.20** | **2.33** | **1.27** | **5.69** | **5.57** | **14.02** | **4.20** | **1.19** | **0.63** | **0.51** | **0.49** | **0.38** | **0.20** | **0.15** | **0.15** |
| **Tc7** | **5.02** | **6.40** | **3.46** | **1.96** | **30.10** | **11.58** | **18.19** | **6.65** | **2.23** | **2.04** | **1.34** | **1.38** | **1.46** | **0.69** | **0.37** | **0.44** |
| **Ts1** | **3.69** | **4.16** | **3.57** | **1.68** | **4.98** | **7.59** | **12.80** | **2.80** | **1.05** | **1.25** | **0.57** | **0.69** | **0.56** | **0.69** | **0.22** | **0.20** |
| **Ts2** | **4.94** | **3.21** | **2.55** | **2.90** | **6.61** | **7.75** | **9.61** | **2.43** | **1.06** | **0.64** | **0.76** | **0.69** | **0.65** | **0.24** | **0.14** | **0.14** |
| **Ts3** | **3.83** | **4.81** | **2.26** | **1.19** | **9.39** | **5.36** | **14.29** | **1.70** | **1.47** | **1.33** | **0.80** | **0.45** | **0.82** | **0.51** | **0.15** | **0.12** |
| **Ts4** | **4.85** | **4.35** | **2.52** | **1.80** | **5.18** | **10.22** | **10.05** | **2.15** | **1.17** | **1.11** | **0.84** | **0.64** | **0.60** | **0.52** | **0.21** | **0.15** |
| **Ts5** | **3.21** | **2.16** | **1.98** | **1.28** | **6.48** | **5.05** | **12.11** | **4.74** | **0.97** | **0.91** | **0.76** | **0.74** | **0.36** | **0.23** | **0.15** | **0.20** |
| **Ts6** | **3.12** | **4.12** | **3.82** | **2.98** | **31.02** | **9.38** | **15.89** | **4.77** | **1.21** | **1.63** | **0.90** | **0.71** | **1.10** | **0.43** | **0.25** | **0.15** |
| **Tsh1** | **2.72** | **2.29** | **2.70** | **1.14** | **14.53** | **7.84** | **15.04** | **11.91** | **1.32** | **1.36** | **1.10** | **0.54** | **1.03** | **0.52** | **0.39** | **0.15** |
| **Tsh2** | **2.29** | **2.16** | **2.76** | **1.49** | **12.70** | **5.67** | **20.32** | **3.72** | **1.00** | **1.15** | **1.31** | **0.67** | **0.53** | **0.45** | **0.34** | **0.16** |
| **Tsh3** | **3.32** | **2.20** | **3.10** | **1.81** | **11.85** | **7.17** | **15.44** | **7.26** | **1.25** | **1.28** | **1.40** | **0.98** | **0.76** | **0.45** | **0.50** | **0.29** |
| **Tsh4** | **2.28** | **2.66** | **2.64** | **1.64** | **11.78** | **9.12** | **15.72** | **7.36** | **0.93** | **1.19** | **1.10** | **0.67** | **0.60** | **0.42** | **0.60** | **0.18** |
| **Tsh5** | **2.52** | **3.13** | **2.61** | **2.41** | **11.70** | **7.43** | **11.50** | **5.82** | **1.15** | **1.78** | **1.06** | **0.80** | **0.43** | **0.54** | **0.28** | **0.17** |
| **Tt1** | **3.62** | **2.03** | **2.72** | **2.74** | **30.07** | **5.42** | **11.04** | **1.11** | **2.80** | **1.42** | **1.18** | **1.23** | **1.39** | **0.53** | **0.41** | **0.00** |
| **Tz1** | **3.70** | **3.15** | **2.87** | **2.28** | **59.43** | **13.62** | **36.72** | **11.10** | **2.73** | **1.86** | **1.84** | **0.91** | **1.47** | **0.69** | **2.26** | **0.29** |
| **Tsc1** | **3.92** | **2.28** | **2.50** | **2.03** | **15.52** | **7.33** | **40.57** | **19.75** | **1.99** | **1.41** | **1.25** | **1.12** | **0.50** | **0.46** | **0.66** | **0.21** |

**Supplementary Table S4**. The shoot and root copper (Cu), and zinc (Zn) uptake in 30 hexaploid wheat genotypes under Control (C), drought (D), salinity (S) and combined drought and salinity (D+S) treatments. Nutrient uptake is presented as µg/plant.

| **Genotype Code** | **Accum Shoot Cu** | | | | **Accum Root Cu** | | | | **Accum Shoot Zn** | | | | **Accum Root Zn** | | | |
| --- | --- | --- | --- | --- | --- | --- | --- | --- | --- | --- | --- | --- | --- | --- | --- | --- |
|  | **C** | **D** | **S** | **D+S** | **C** | **D** | **S** | **D+S** | **C** | **D** | **S** | **D+S** | **C** | **D** | **S** | **D+S** |
| **Ta1** | **1.17** | **1.39** | **0.99** | **0.87** | **2.19** | **0.75** | **0.99** | **0.73** | **4.62** | **1.02** | **0.92** | **0.80** | **4.38** | **0.61** | **0.89** | **0.41** |
| **Ta2** | **0.94** | **0.82** | **0.08** | **0.63** | **2.05** | **0.74** | **0.89** | **0.73** | **3.38** | **1.64** | **0.08** | **1.40** | **3.39** | **0.83** | **0.69** | **1.06** |
| **Ta3** | **1.06** | **1.15** | **0.91** | **0.96** | **1.48** | **1.14** | **0.80** | **0.74** | **2.04** | **1.65** | **1.83** | **1.25** | **2.18** | **1.54** | **0.97** | **1.11** |
| **Ta4** | **1.21** | **0.80** | **0.52** | **0.60** | **2.30** | **0.79** | **0.50** | **0.52** | **5.18** | **1.09** | **0.71** | **0.76** | **5.89** | **0.79** | **0.56** | **0.78** |
| **Ta5** | **1.49** | **0.53** | **0.29** | **0.21** | **1.22** | **0.49** | **0.14** | **0.15** | **2.19** | **1.06** | **0.87** | **0.63** | **1.11** | **0.35** | **0.14** | **0.00** |
| **Ta6** | **0.97** | **0.42** | **0.79** | **0.91** | **3.19** | **0.71** | **1.23** | **4.69** | **4.57** | **1.46** | **2.11** | **1.52** | **2.15** | **1.16** | **1.16** | **1.92** |
| **Ta7** | **0.73** | **0.37** | **0.78** | **0.89** | **2.78** | **0.58** | **1.57** | **3.30** | **3.28** | **1.54** | **1.92** | **1.05** | **3.18** | **0.77** | **2.51** | **1.25** |
| **Ta8** | **0.77** | **0.29** | **0.69** | **0.70** | **3.48** | **1.10** | **1.70** | **4.80** | **4.09** | **1.40** | **1.31** | **1.46** | **3.27** | **1.01** | **1.42** | **3.03** |
| **Ta9** | **0.94** | **0.42** | **0.90** | **0.75** | **3.39** | **1.07** | **2.20** | **5.22** | **5.04** | **1.73** | **2.29** | **1.30** | **3.10** | **1.15** | **1.83** | **2.53** |
| **Tc1** | **1.59** | **0.82** | **0.55** | **0.29** | **0.90** | **0.53** | **0.30** | **0.14** | **2.74** | **0.68** | **0.83** | **0.71** | **0.81** | **0.30** | **0.15** | **0.14** |
| **Tc2** | **1.21** | **1.02** | **0.31** | **0.24** | **0.82** | **0.39** | **0.13** | **0.22** | **2.25** | **1.16** | **0.69** | **0.67** | **0.90** | **0.55** | **0.06** | **0.22** |
| **Tc3** | **0.94** | **0.56** | **0.36** | **0.29** | **0.72** | **0.44** | **0.14** | **0.16** | **1.31** | **1.13** | **0.50** | **0.43** | **0.80** | **0.15** | **0.00** | **0.00** |
| **Tc4** | **0.92** | **0.84** | **0.21** | **0.36** | **0.49** | **0.08** | **0.22** | **0.24** | **0.69** | **0.98** | **0.85** | **0.58** | **0.49** | **0.15** | **0.22** | **0.16** |
| **Tc5** | **1.32** | **0.44** | **0.42** | **0.37** | **0.87** | **0.48** | **0.29** | **0.24** | **1.62** | **0.44** | **0.55** | **0.60** | **2.15** | **0.08** | **0.43** | **0.24** |
| **Tc6** | **0.82** | **0.69** | **0.36** | **0.14** | **0.54** | **0.50** | **0.22** | **0.15** | **0.97** | **0.63** | **0.65** | **0.35** | **0.23** | **0.00** | **0.07** | **0.07** |
| **Tc7** | **1.12** | **1.09** | **0.92** | **0.44** | **6.59** | **4.22** | **3.98** | **1.46** | **4.88** | **4.07** | **2.19** | **2.25** | **2.93** | **2.38** | **2.95** | **2.12** |
| **Ts1** | **0.60** | **1.18** | **0.21** | **0.23** | **0.42** | **0.84** | **0.22** | **0.13** | **0.90** | **1.18** | **0.64** | **0.61** | **0.21** | **0.38** | **0.14** | **0.07** |
| **Ts2** | **1.06** | **0.93** | **0.48** | **0.31** | **0.58** | **0.95** | **0.36** | **0.21** | **1.06** | **0.64** | **0.90** | **0.53** | **0.29** | **0.12** | **0.36** | **0.07** |
| **Ts3** | **0.66** | **1.19** | **0.44** | **0.22** | **0.89** | **0.77** | **0.22** | **0.12** | **1.92** | **1.12** | **0.66** | **0.37** | **0.96** | **0.26** | **0.15** | **0.18** |
| **Ts4** | **0.86** | **1.31** | **0.39** | **0.32** | **0.46** | **0.78** | **0.21** | **0.15** | **1.72** | **1.38** | **1.17** | **0.77** | **0.27** | **0.43** | **0.36** | **0.15** |
| **Ts5** | **0.67** | **0.42** | **0.38** | **0.20** | **0.58** | **0.15** | **0.15** | **0.14** | **0.90** | **0.77** | **0.76** | **0.47** | **0.29** | **0.08** | **0.08** | **0.14** |
| **Ts6** | **0.57** | **0.78** | **0.97** | **0.85** | **4.88** | **4.54** | **3.23** | **1.06** | **2.91** | **3.12** | **2.02** | **1.49** | **2.48** | **2.31** | **2.04** | **1.29** |
| **Tsh1** | **0.56** | **0.50** | **0.88** | **0.25** | **2.61** | **2.96** | **2.05** | **0.90** | **3.20** | **2.36** | **2.34** | **0.99** | **1.66** | **0.87** | **2.05** | **1.05** |
| **Tsh2** | **0.57** | **0.58** | **0.69** | **0.30** | **1.76** | **1.26** | **3.27** | **0.57** | **2.00** | **2.08** | **2.15** | **1.19** | **0.71** | **1.08** | **2.50** | **0.89** |
| **Tsh3** | **0.59** | **0.35** | **0.74** | **0.33** | **1.74** | **1.34** | **2.49** | **0.95** | **1.92** | **1.49** | **1.62** | **1.23** | **1.06** | **0.75** | **1.59** | **0.73** |
| **Tsh4** | **0.57** | **0.49** | **0.67** | **0.22** | **2.64** | **2.44** | **2.47** | **0.55** | **2.71** | **3.36** | **2.39** | **1.27** | **1.36** | **1.53** | **2.14** | **0.92** |
| **Tsh5** | **0.65** | **0.57** | **0.71** | **0.66** | **2.09** | **3.07** | **2.41** | **0.87** | **2.59** | **2.78** | **1.91** | **1.61** | **0.94** | **2.07** | **1.70** | **0.78** |
| **Tt1** | **0.89** | **0.41** | **0.59** | **0.69** | **2.94** | **0.83** | **1.10** | **0.26** | **4.37** | **1.49** | **1.91** | **1.03** | **3.84** | **2.56** | **2.14** | **0.09** |
| **Tz1** | **0.74** | **0.48** | **0.74** | **0.59** | **3.91** | **1.45** | **3.84** | **3.60** | **4.88** | **2.02** | **2.06** | **1.24** | **4.32** | **2.28** | **2.71** | **1.62** |
| **Tsc1** | **0.89** | **0.47** | **0.44** | **0.42** | **3.70** | **1.39** | **3.77** | **4.05** | **4.88** | **2.44** | **1.91** | **1.68** | **2.49** | **2.08** | **2.46** | **1.14** |
