# Supplementary figures and images for: Nutrient uptake under combined drought and salinity stress in hexaploid wheat species
Source: Front Plant Sci. 2025 Nov 10;16:1682258. doi: 10.3389/fpls.2025.1682258 (PMC12641000; doi:10.3389/fpls.2025.1682258)

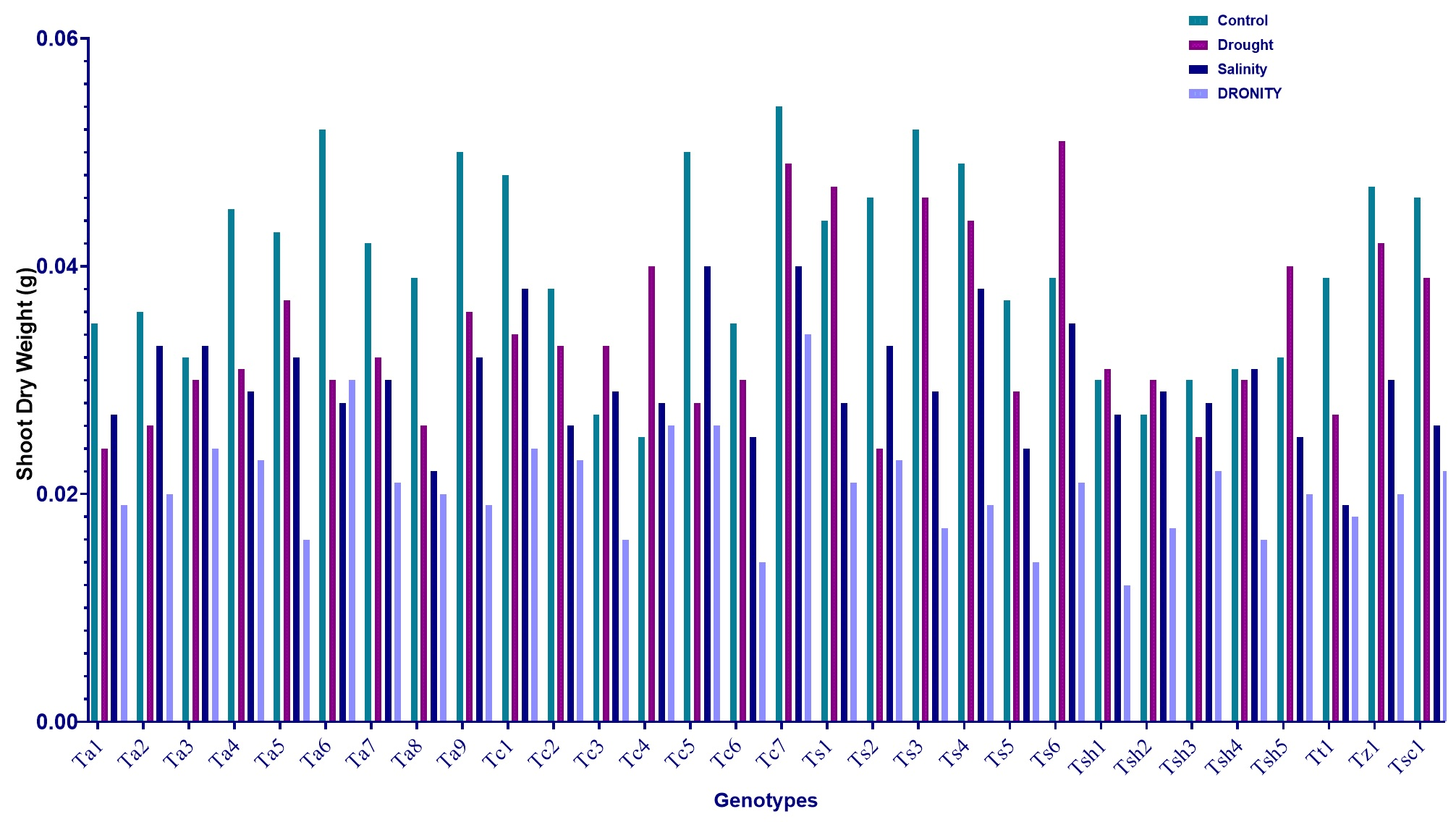

Supplement: Supplementary file 1 [file SupplementaryFile1.zip › Revised Supplementary Files/Figure S1 Shoot Dry Weight300.png]

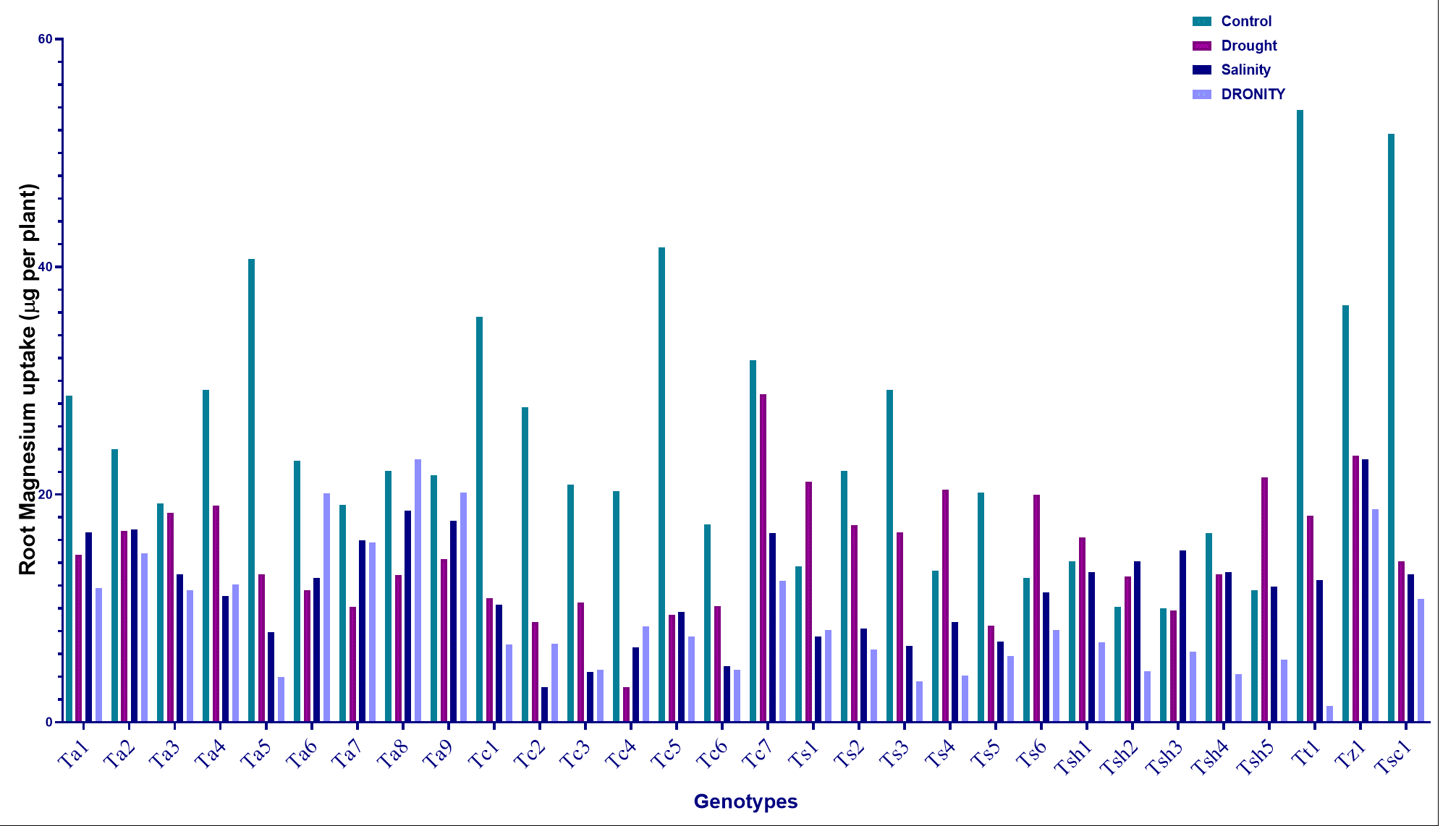

Supplement: Supplementary file 1 [file SupplementaryFile1.zip › Revised Supplementary Files/Figure S10 Root Magnesium uptake300.png]

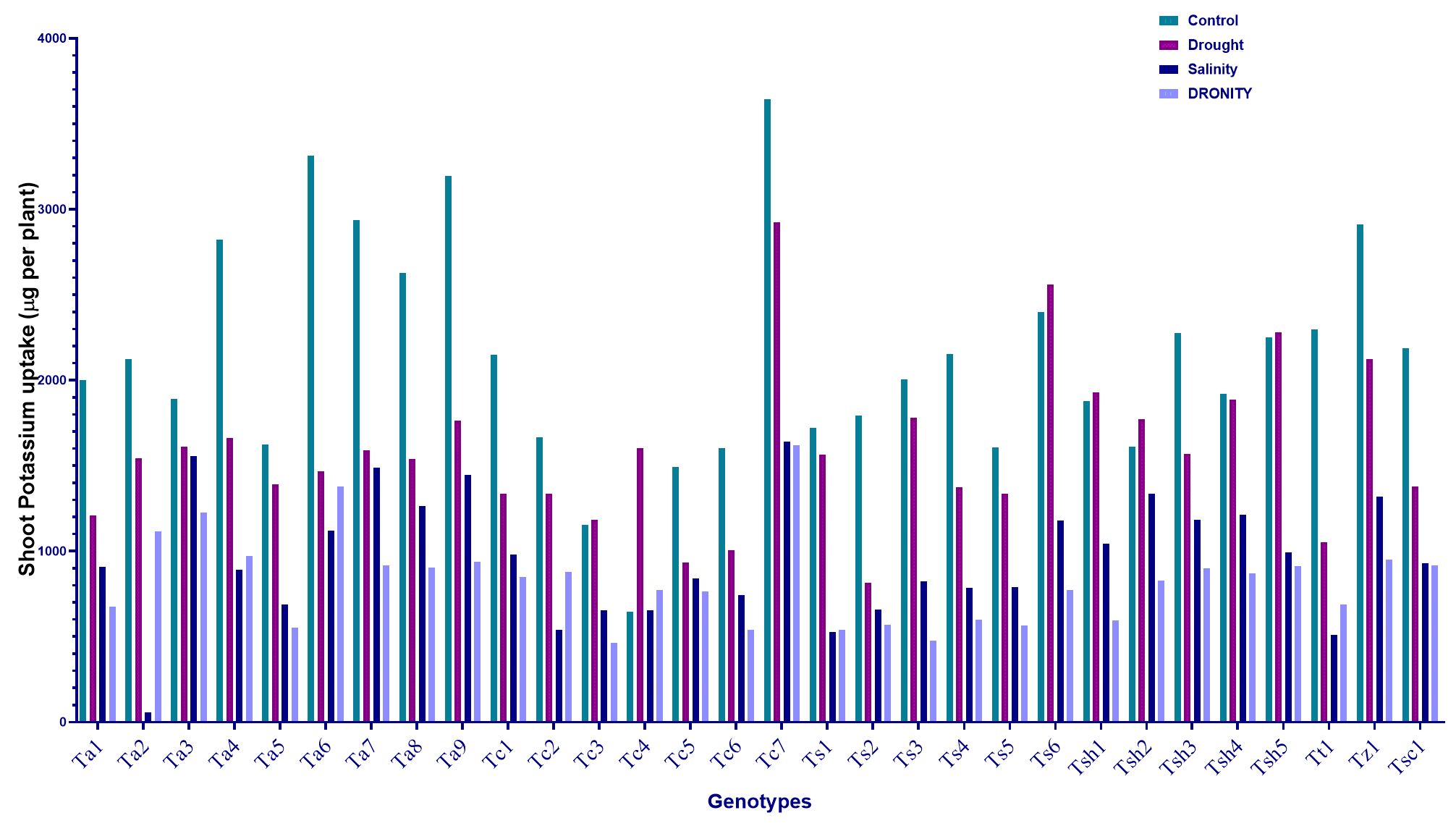

Supplement: Supplementary file 1 [file SupplementaryFile1.zip › Revised Supplementary Files/Figure S11 Shoot Potassium uptake300.png]

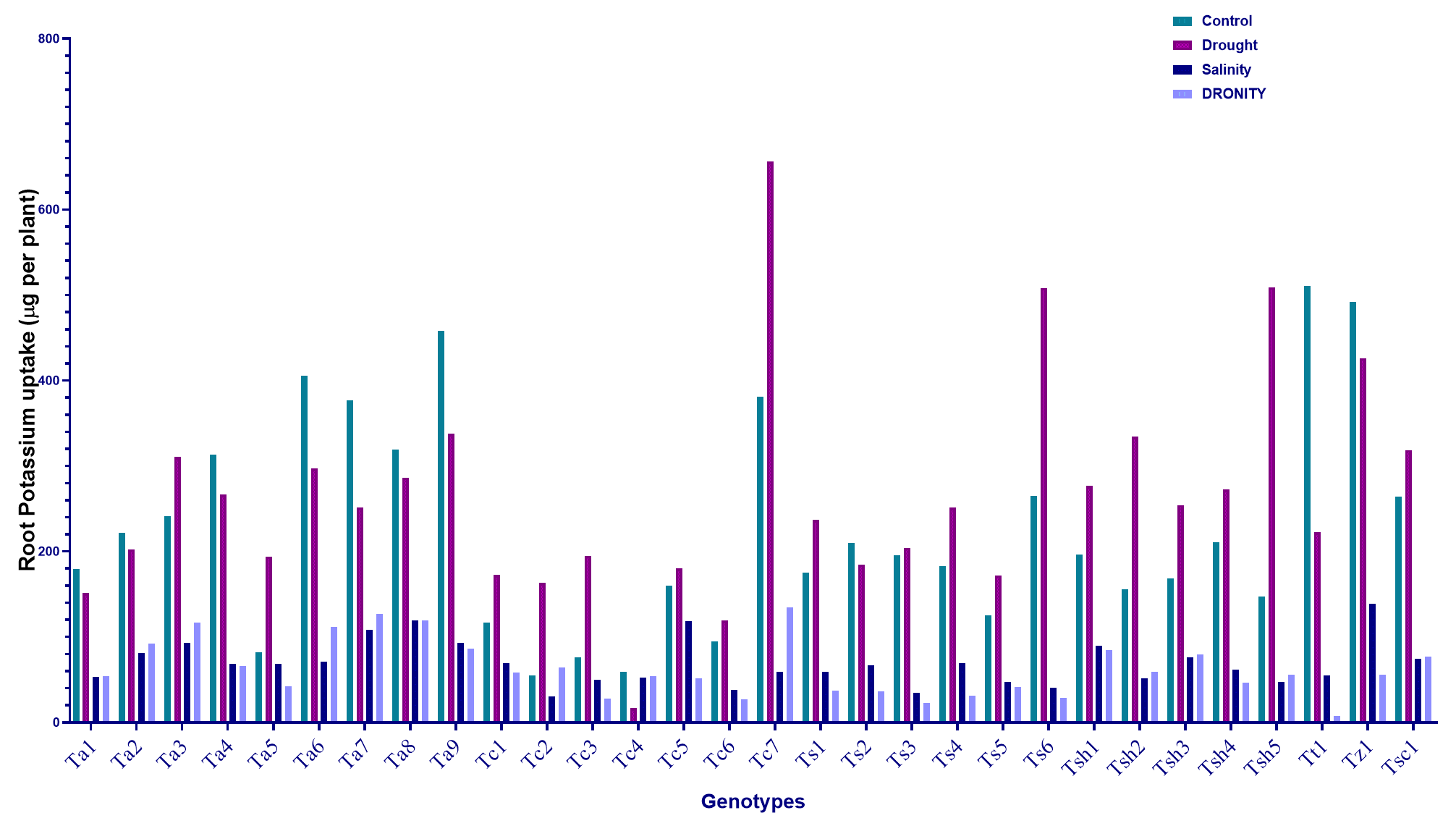

Supplement: Supplementary file 1 [file SupplementaryFile1.zip › Revised Supplementary Files/Figure S12 Root Potassium uptake300.png]

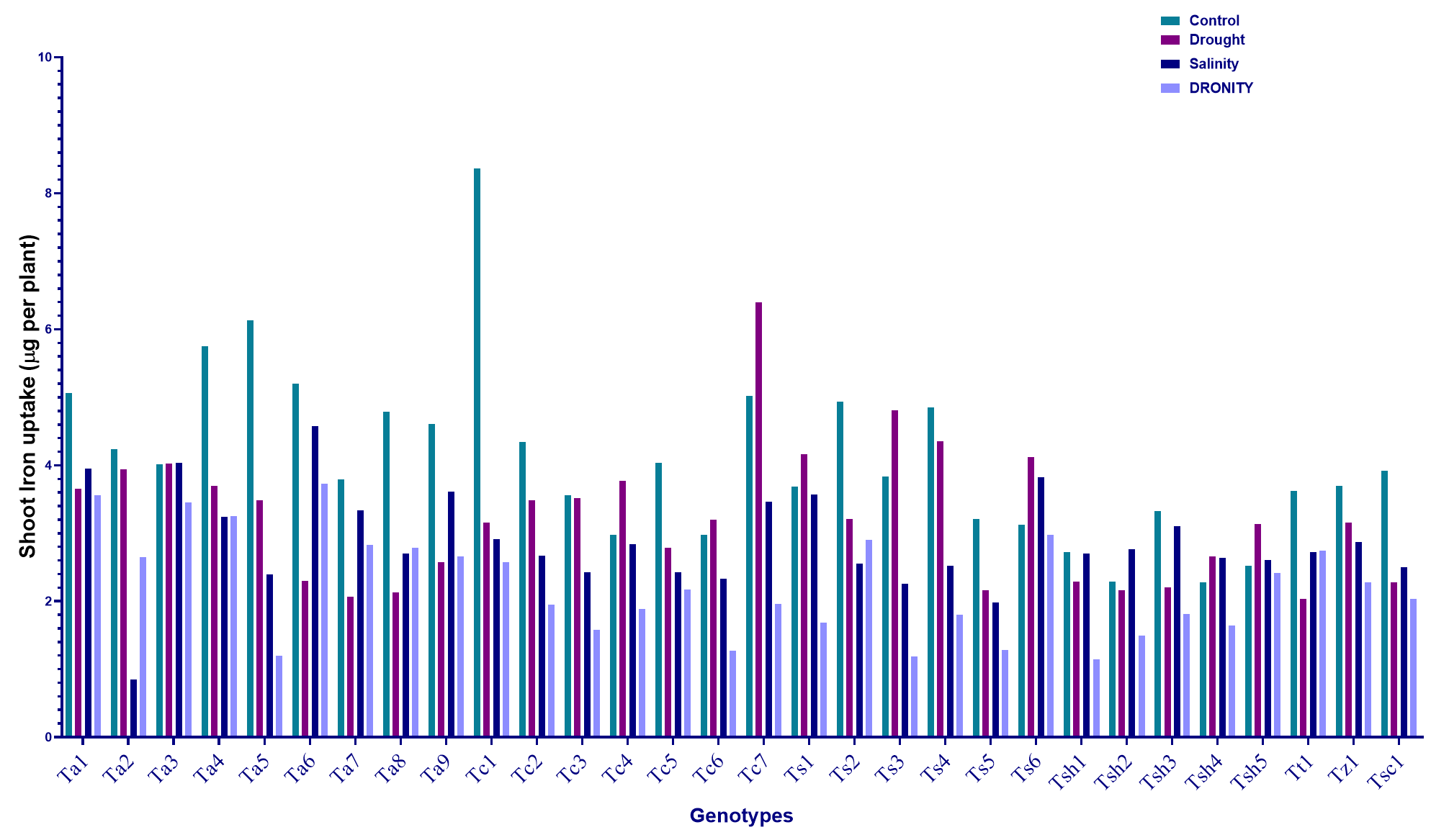

Supplement: Supplementary file 1 [file SupplementaryFile1.zip › Revised Supplementary Files/Figure S13 Shoot Iron uptake300.png]

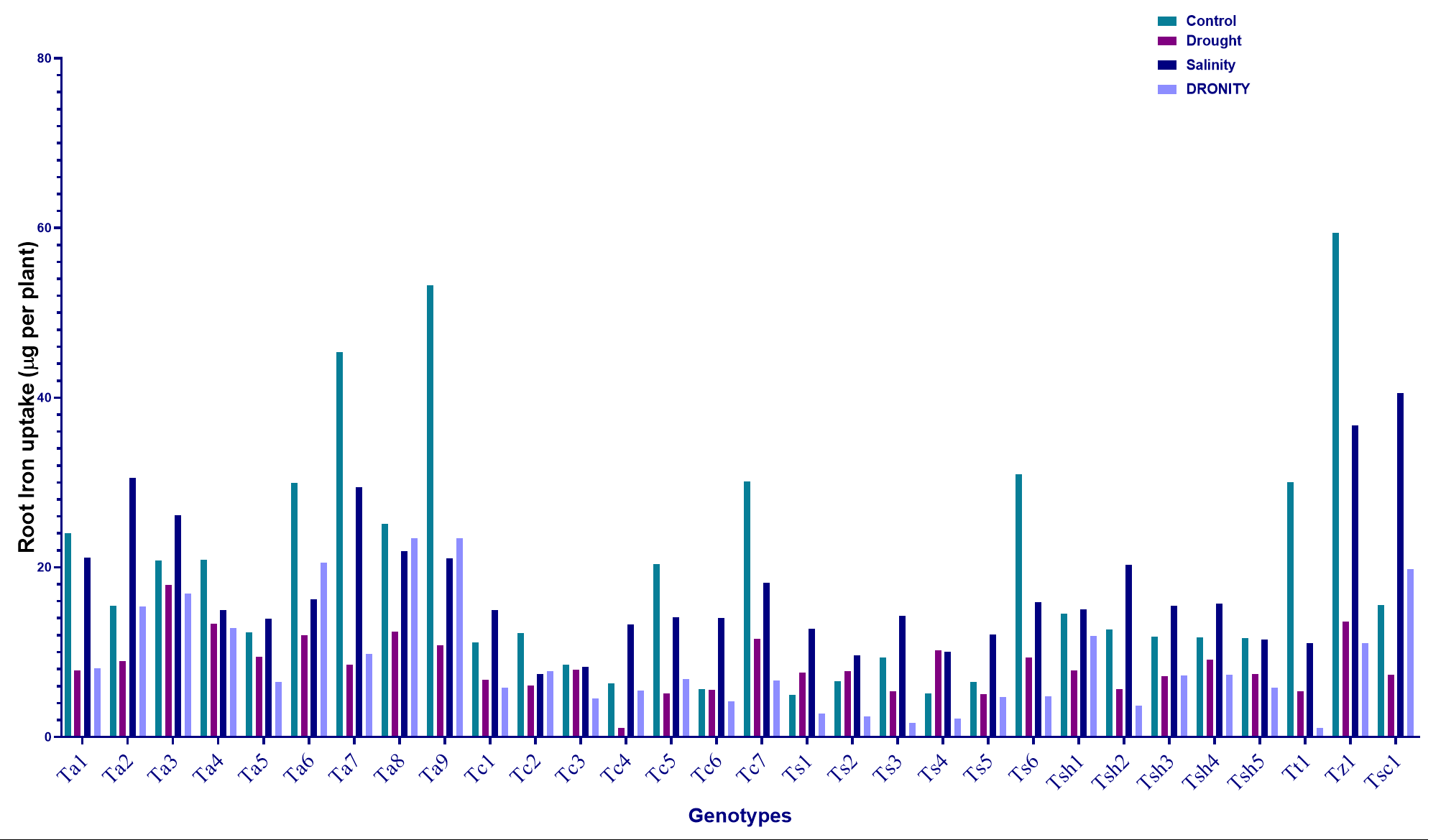

Supplement: Supplementary file 1 [file SupplementaryFile1.zip › Revised Supplementary Files/Figure S14 Root Iron uptake300.png]

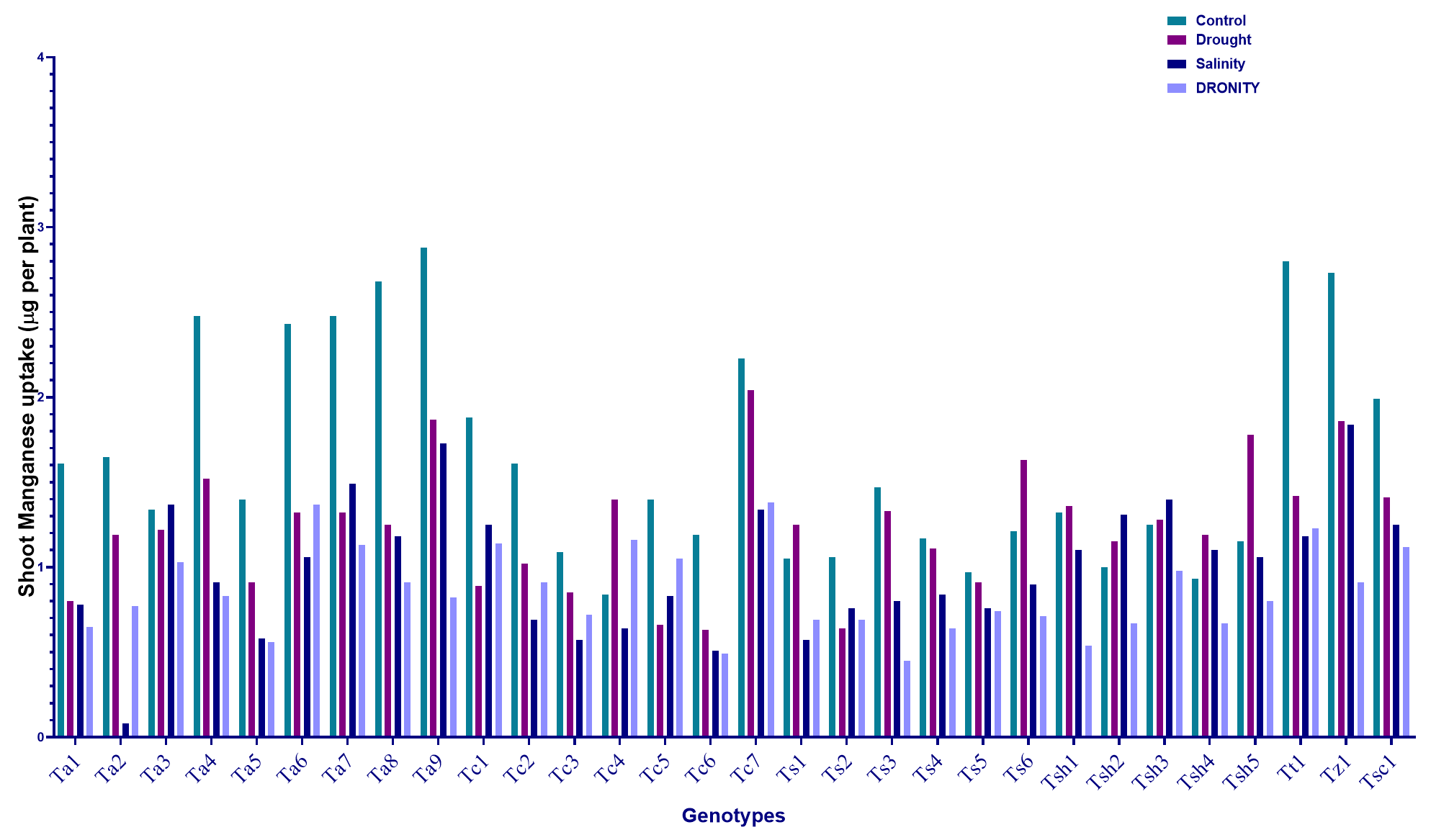

Supplement: Supplementary file 1 [file SupplementaryFile1.zip › Revised Supplementary Files/Figure S15 Shoot Manganese uptake300.png]

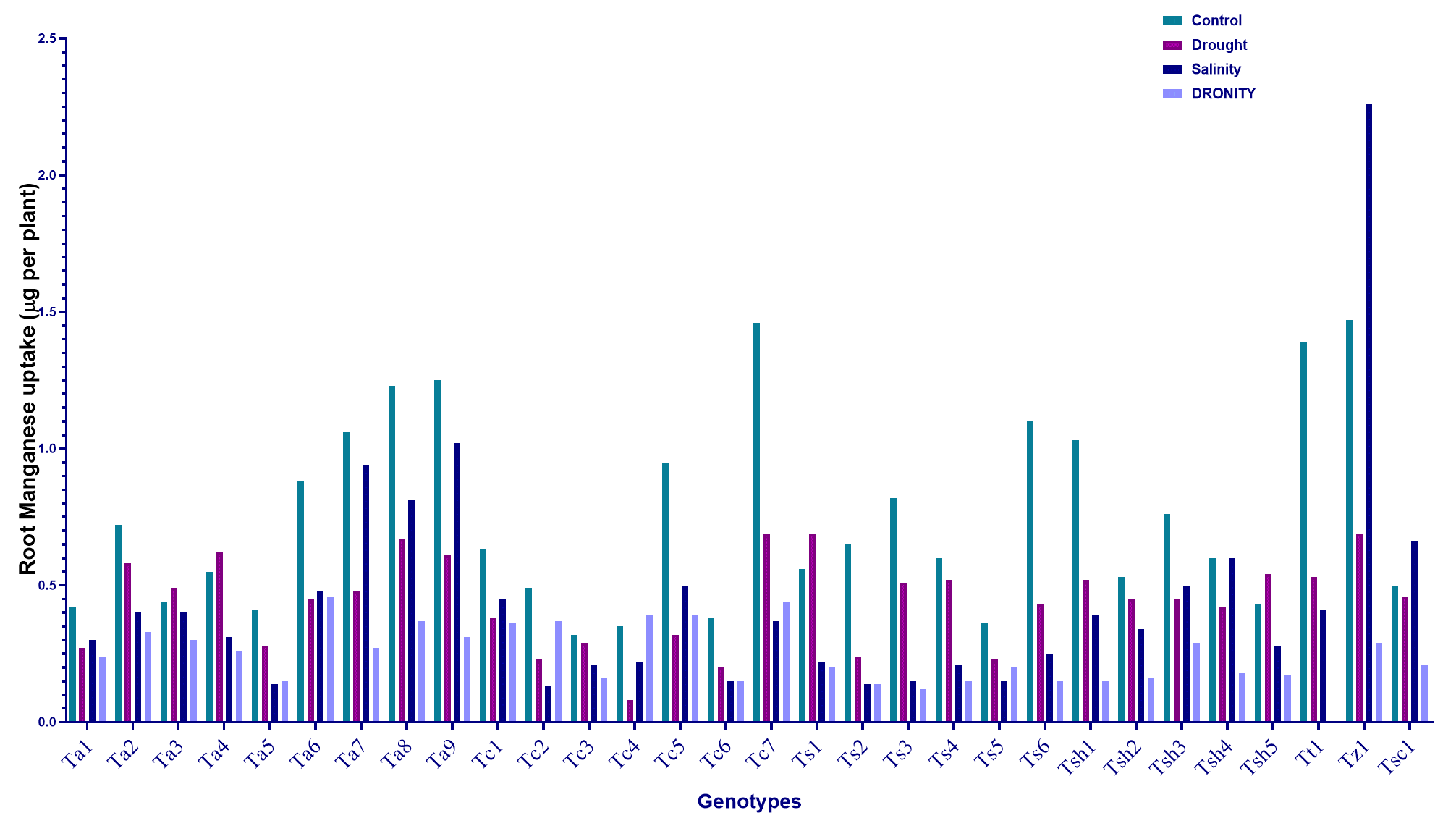

Supplement: Supplementary file 1 [file SupplementaryFile1.zip › Revised Supplementary Files/Figure S16 Root Manganese uptake300.png]

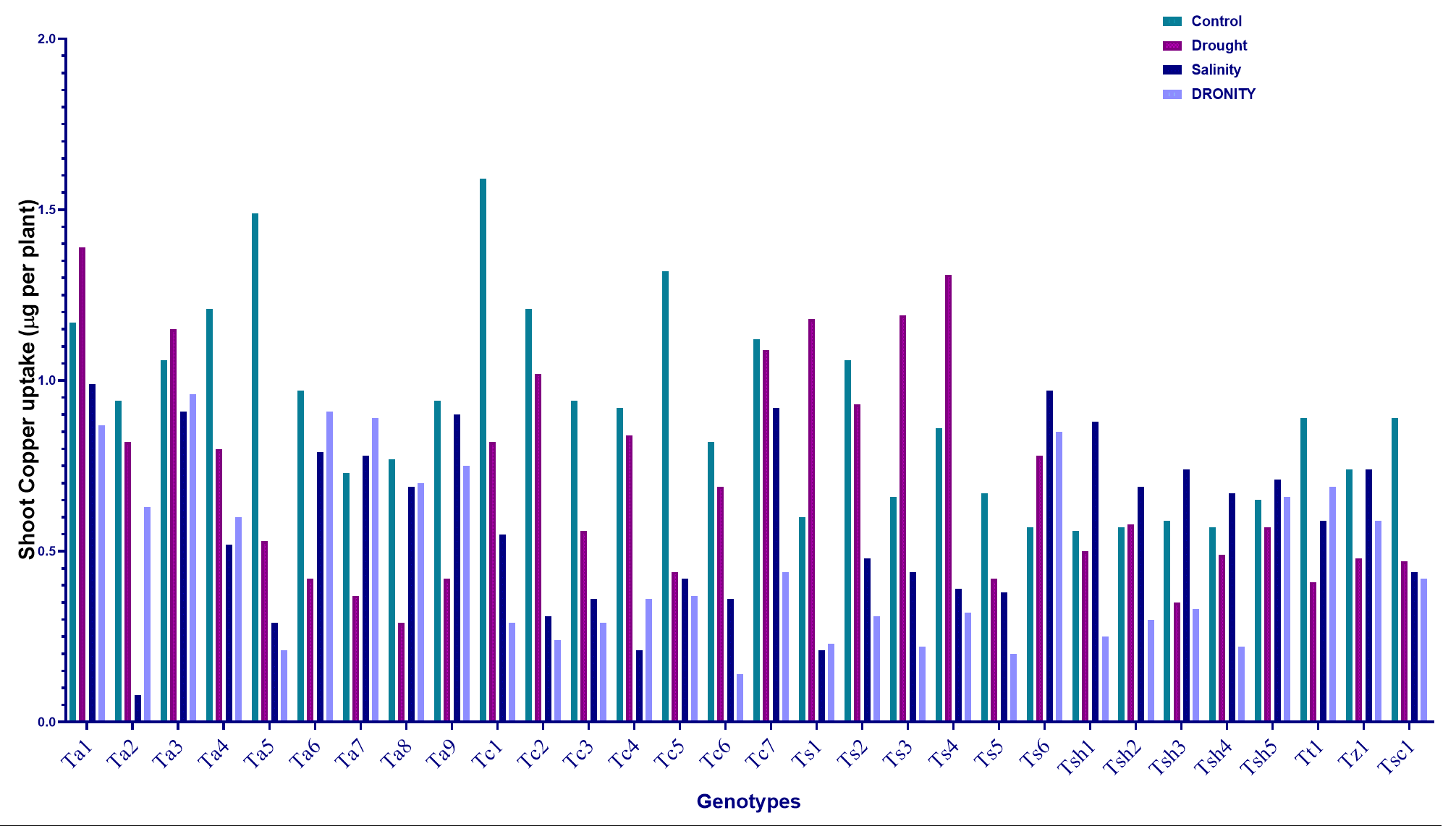

Supplement: Supplementary file 1 [file SupplementaryFile1.zip › Revised Supplementary Files/Figure S17 Shoot Copper uptake300.png]

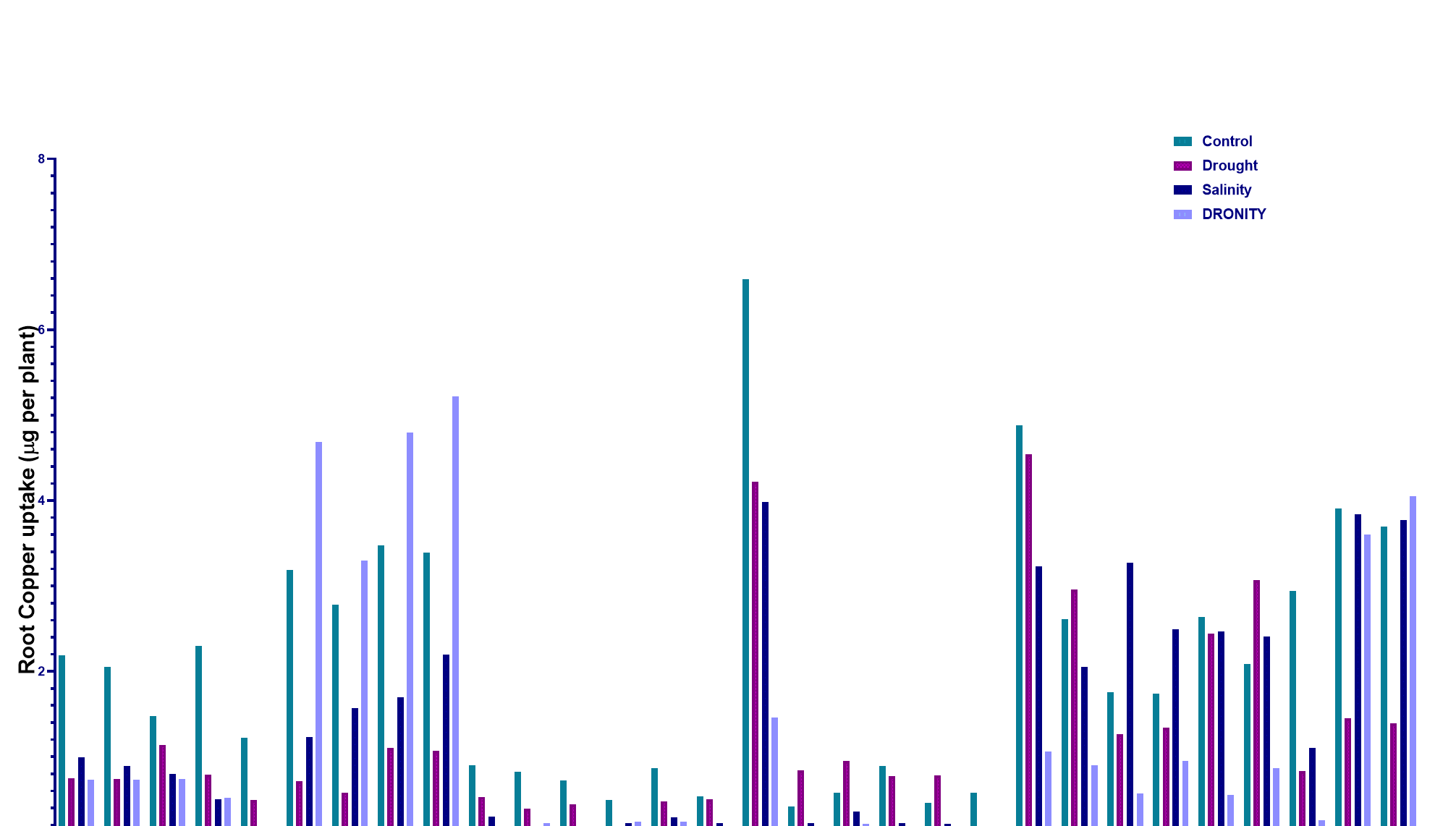

Supplement: Supplementary file 1 [file SupplementaryFile1.zip › Revised Supplementary Files/Figure S18 Root Copper uptake300.png]

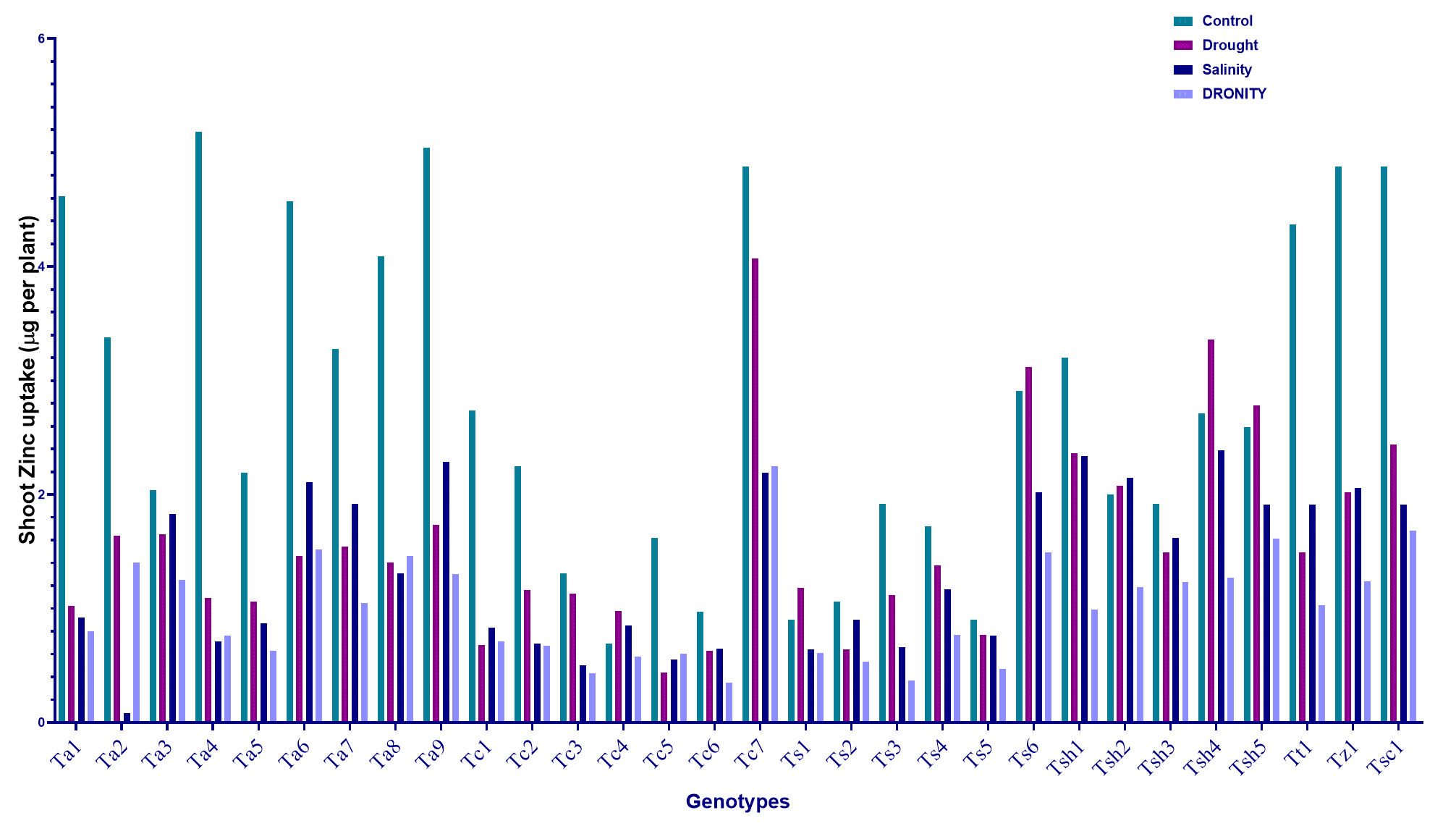

Supplement: Supplementary file 1 [file SupplementaryFile1.zip › Revised Supplementary Files/Figure S19 Shoot Zinc uptake300.png]

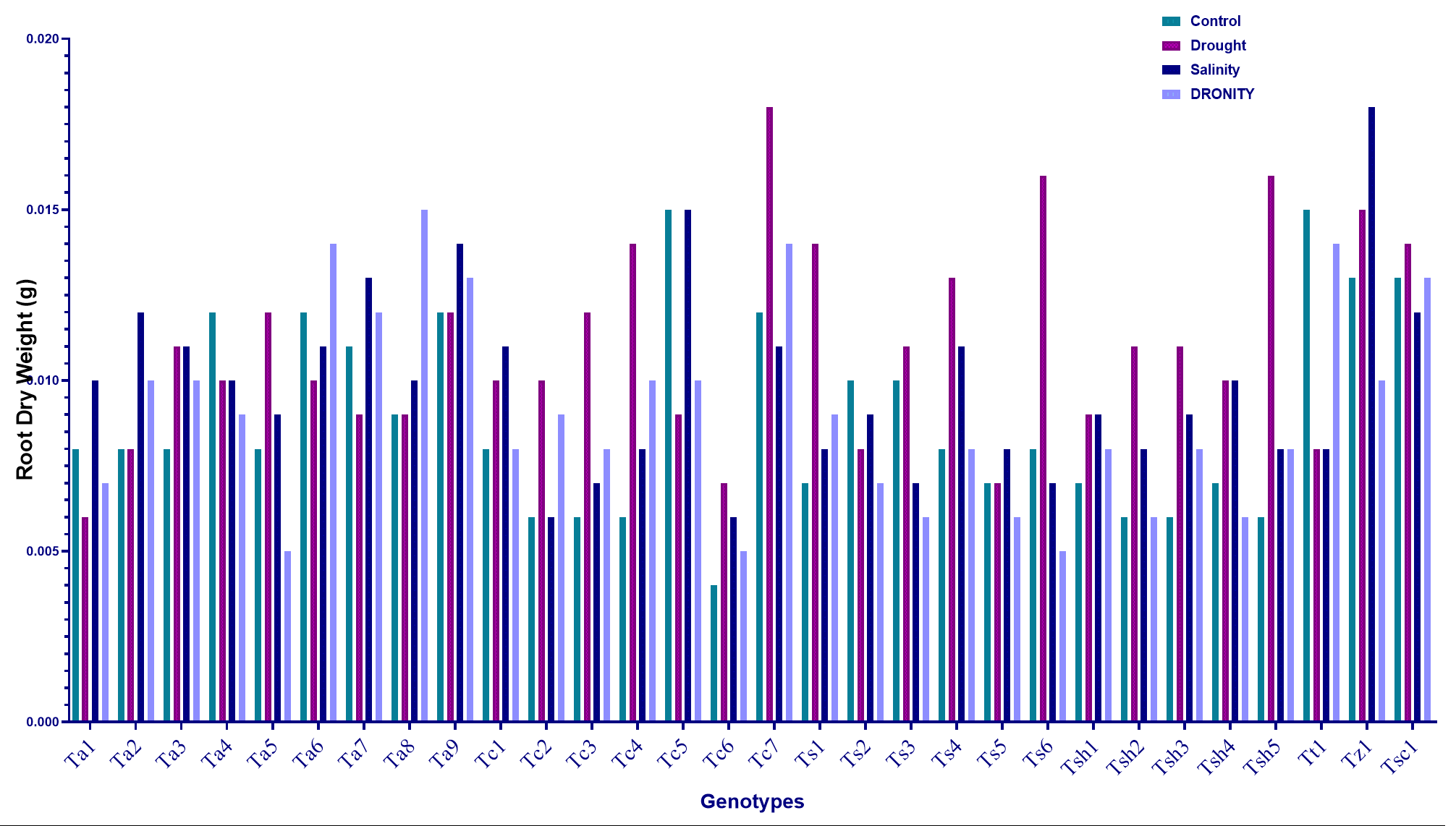

Supplement: Supplementary file 1 [file SupplementaryFile1.zip › Revised Supplementary Files/Figure S2 Root Dry Weight300.png]

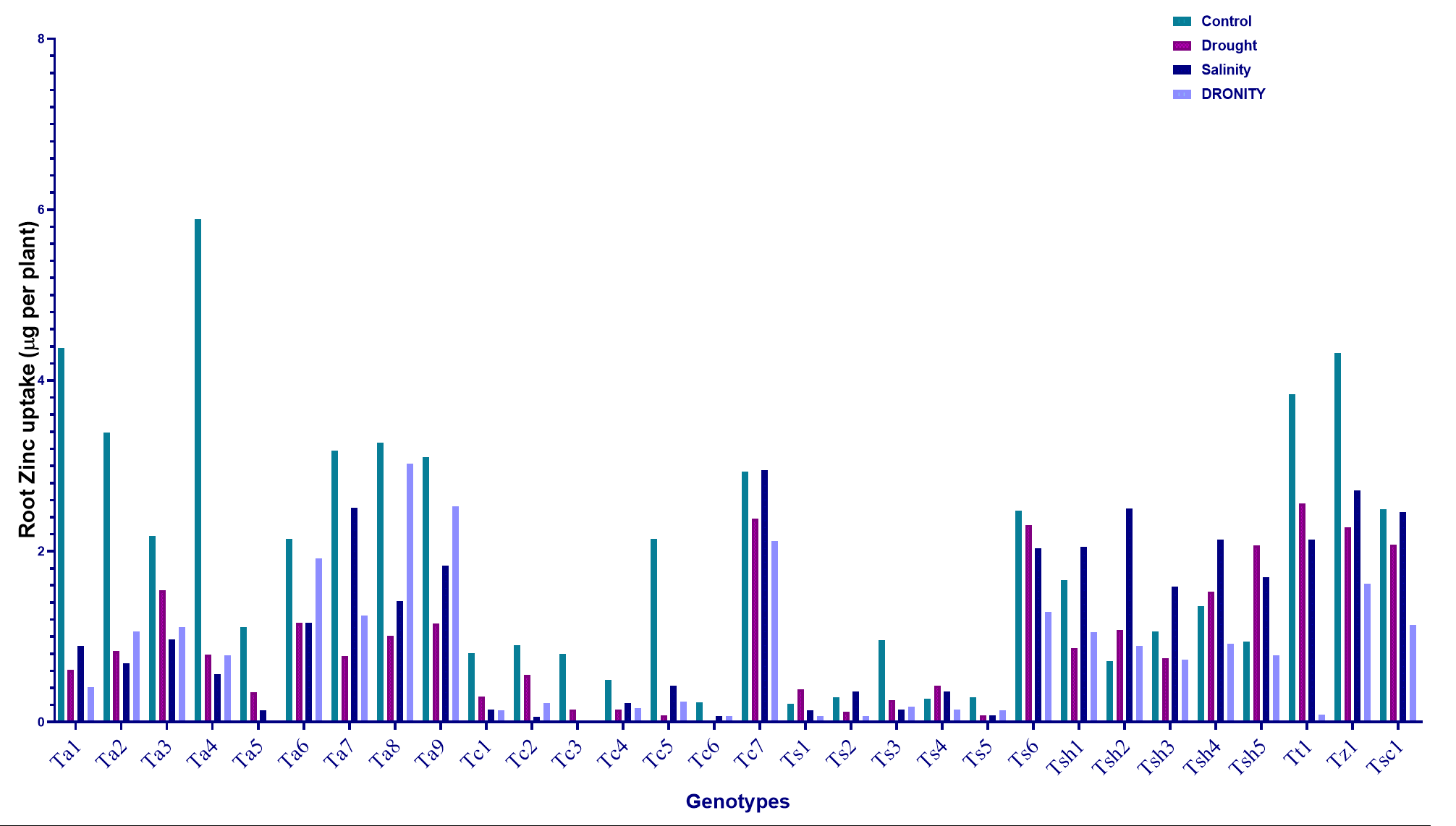

Supplement: Supplementary file 1 [file SupplementaryFile1.zip › Revised Supplementary Files/Figure S20 Root Zinc uptake300.png]

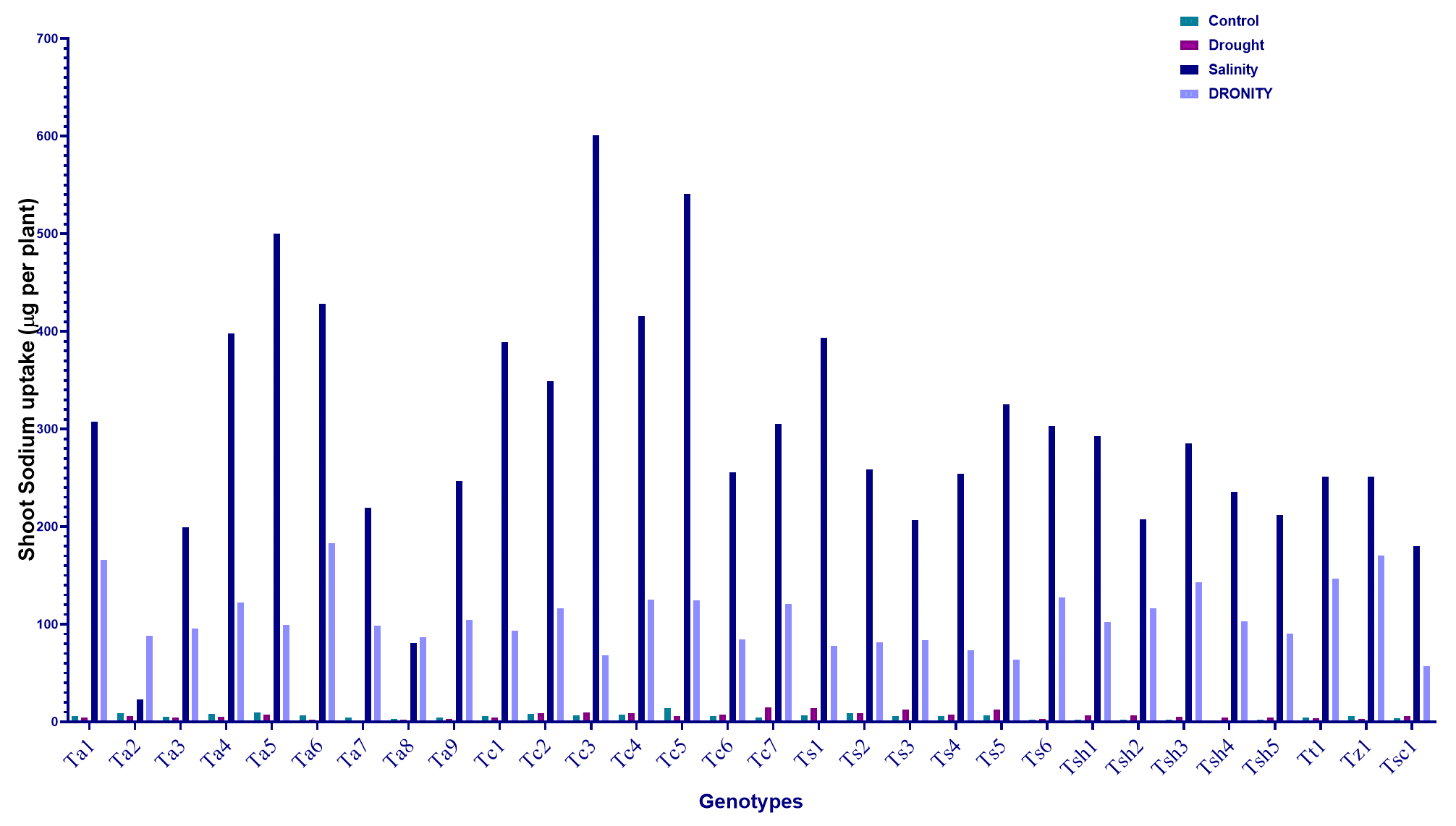

Supplement: Supplementary file 1 [file SupplementaryFile1.zip › Revised Supplementary Files/Figure S3 Shoot Sodium uptake300.png]

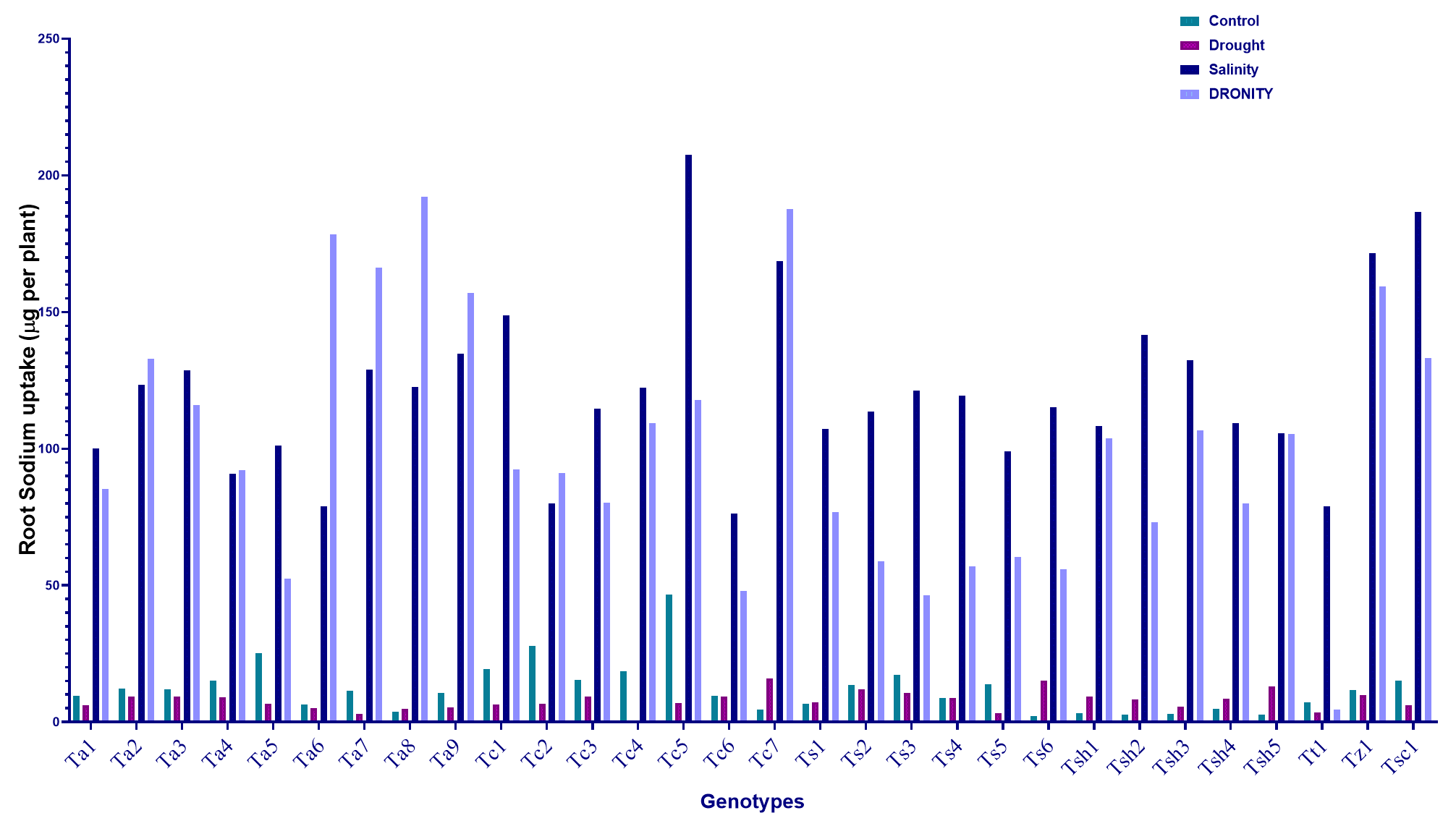

Supplement: Supplementary file 1 [file SupplementaryFile1.zip › Revised Supplementary Files/Figure S4 Root Sodium uptake300.png]

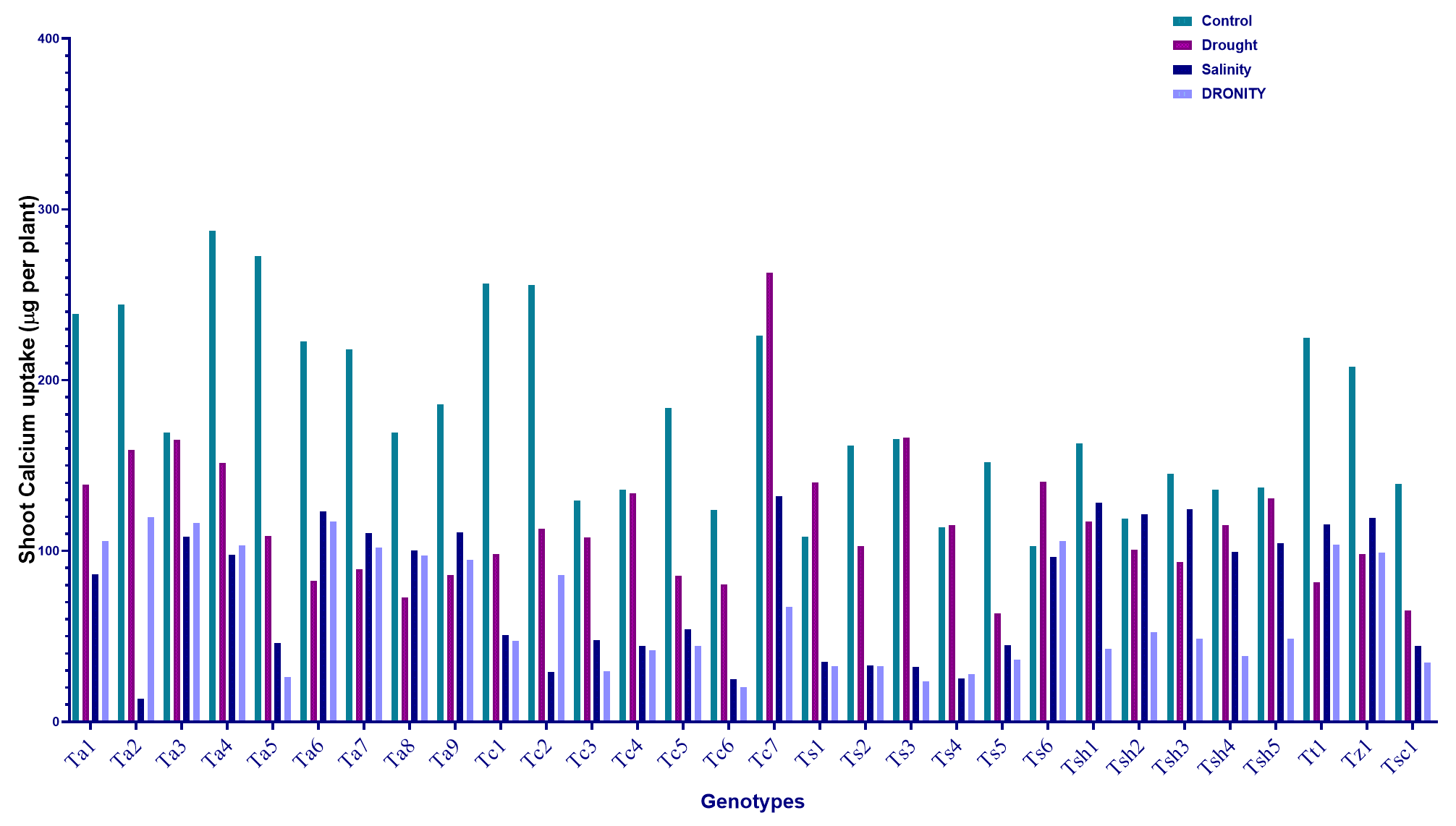

Supplement: Supplementary file 1 [file SupplementaryFile1.zip › Revised Supplementary Files/Figure S5 Shoot Calcium uptake300.png]

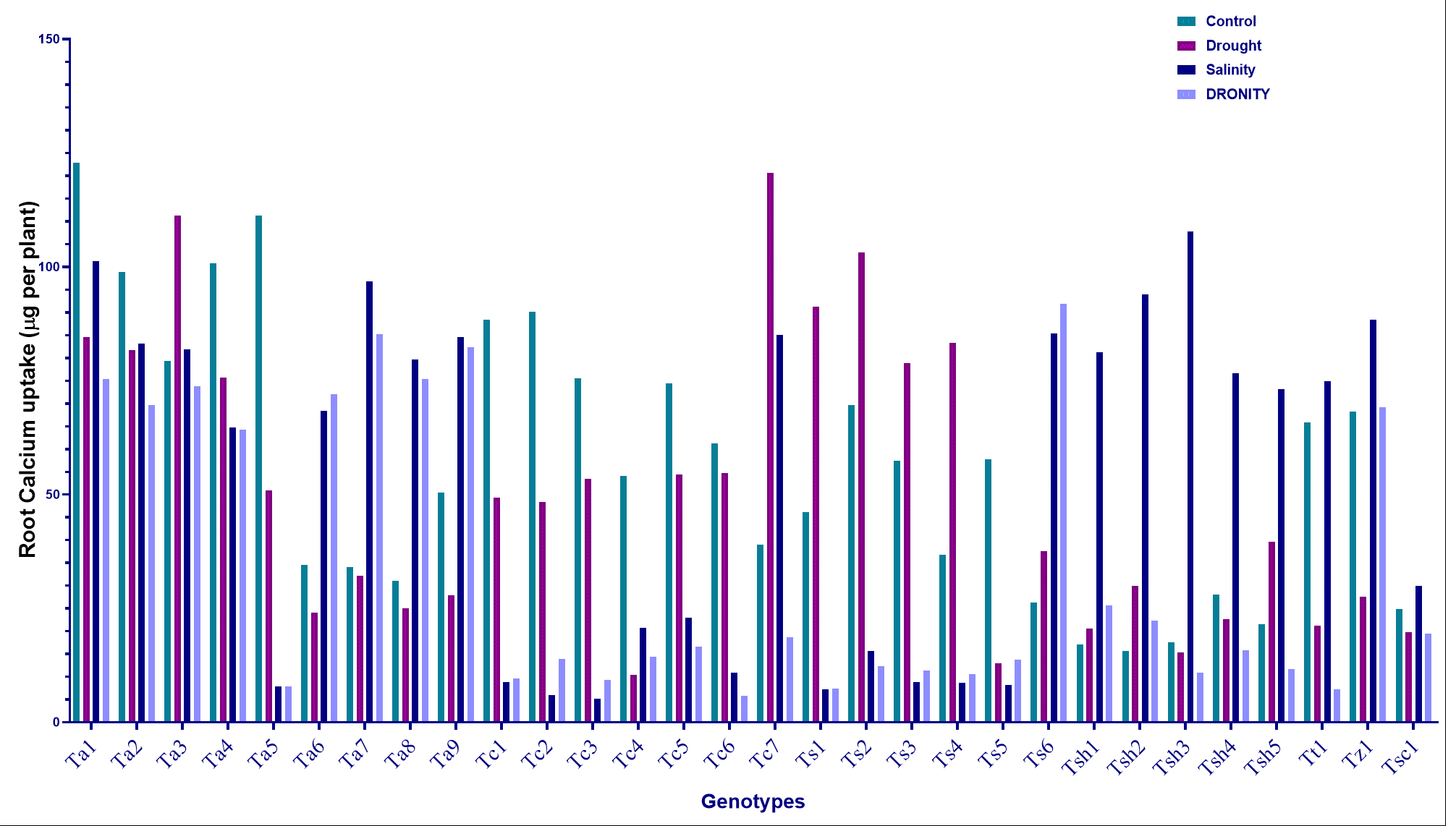

Supplement: Supplementary file 1 [file SupplementaryFile1.zip › Revised Supplementary Files/Figure S6 Root Calcium uptake300.png]

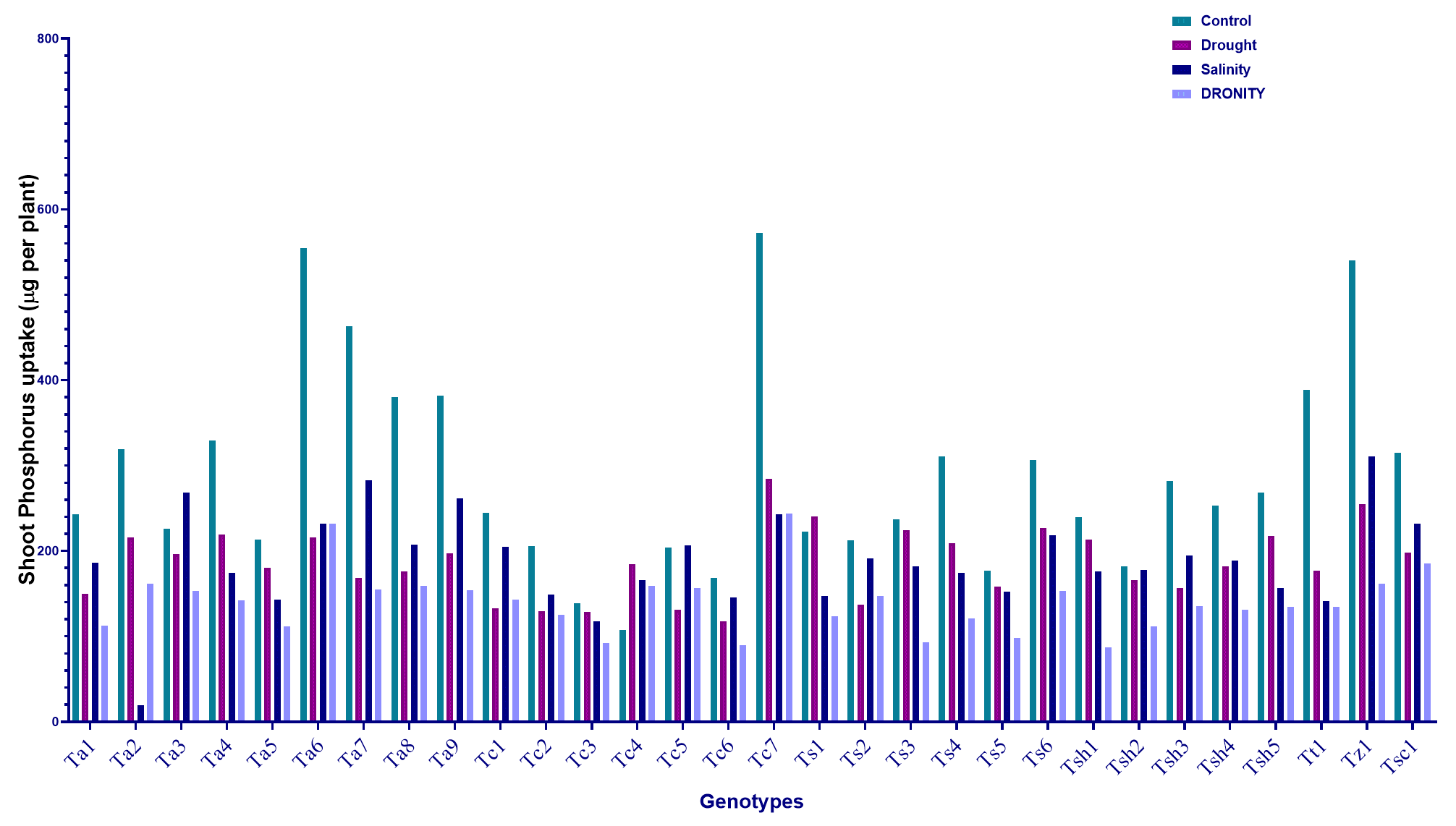

Supplement: Supplementary file 1 [file SupplementaryFile1.zip › Revised Supplementary Files/Figure S7 Shoot Phosphorus uptake300.png]

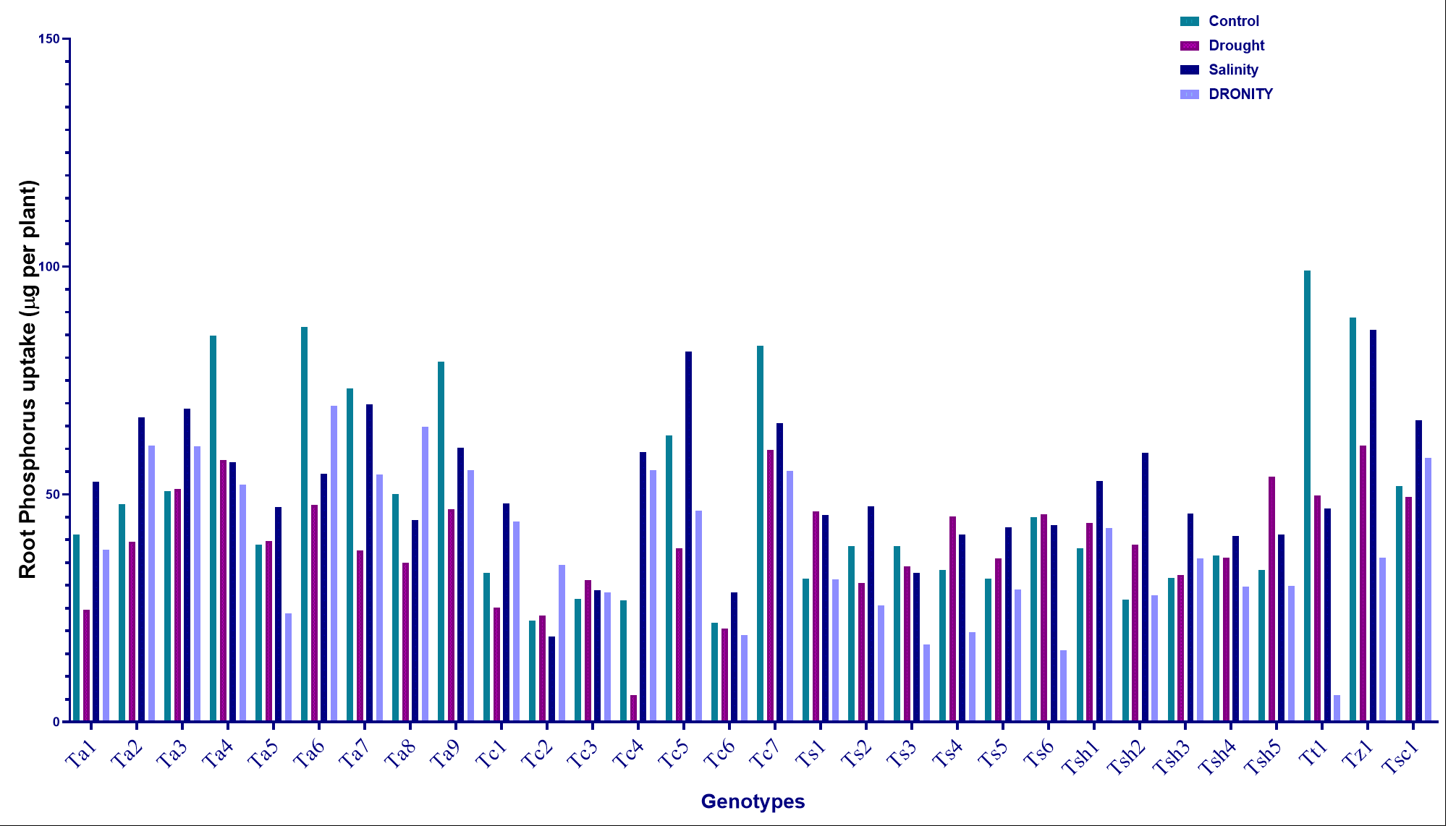

Supplement: Supplementary file 1 [file SupplementaryFile1.zip › Revised Supplementary Files/Figure S8 Root Phosphorus uptake300.png]

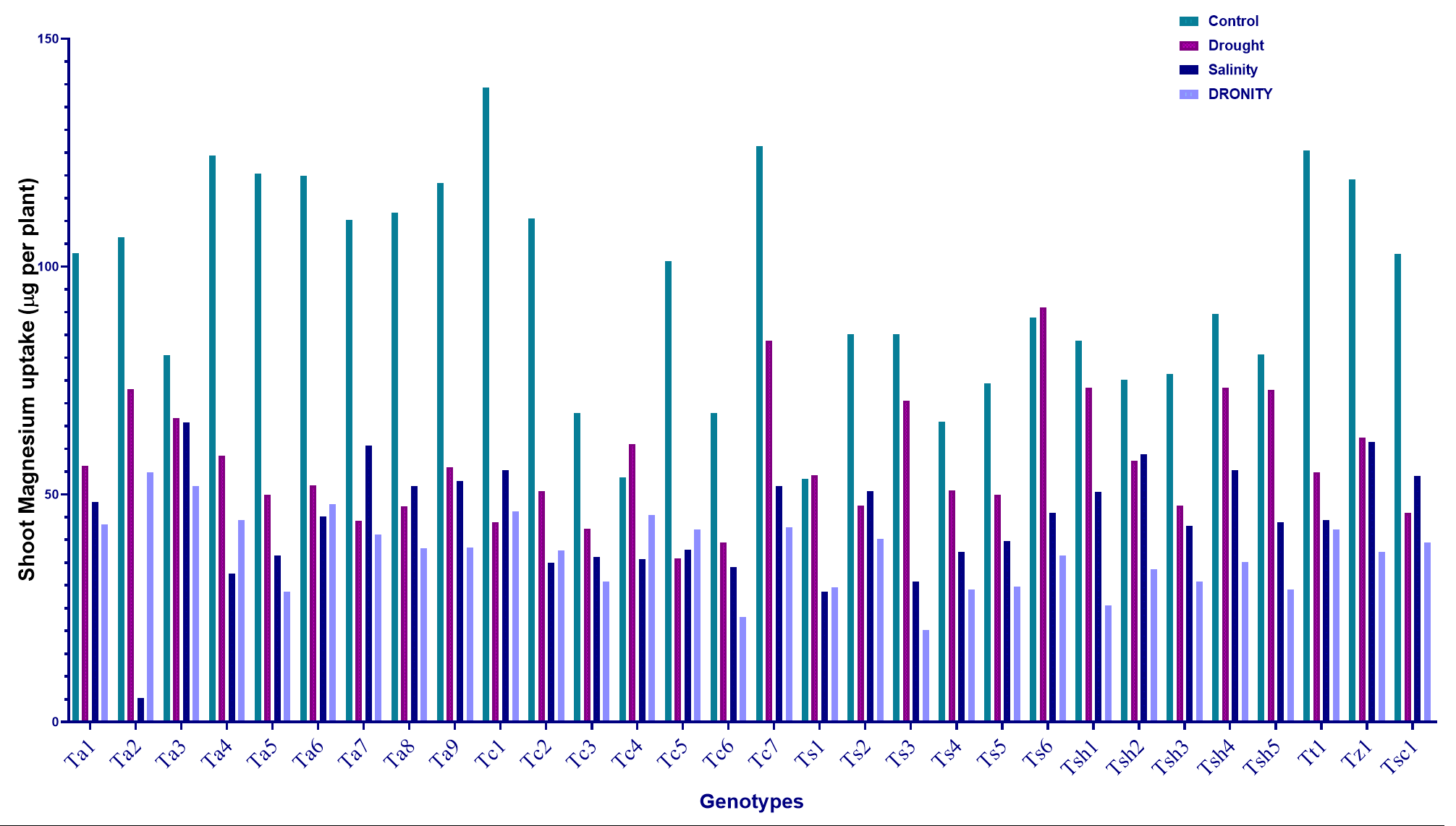

Supplement: Supplementary file 1 [file SupplementaryFile1.zip › Revised Supplementary Files/Figure S9 Shoot Magnesium uptake300.png]
